# Associations between the orexin (hypocretin) receptor 2 gene polymorphism Val308Ile and nicotine dependence in genome-wide and subsequent association studies

**DOI:** 10.1186/s13041-015-0142-x

**Published:** 2015-08-20

**Authors:** Daisuke Nishizawa, Shinya Kasai, Junko Hasegawa, Naomi Sato, Hidetaka Yamada, Fumihiko Tanioka, Makoto Nagashima, Ryoji Katoh, Yasuo Satoh, Megumi Tagami, Hiroshi Ujike, Norio Ozaki, Toshiya Inada, Nakao Iwata, Ichiro Sora, Masaomi Iyo, Mitsuhiko Yamada, Naoki Kondo, Moo-Jun Won, Nobuya Naruse, Kumi Uehara-Aoyama, Masanari Itokawa, Kazutaka Ohi, Ryota Hashimoto, Kumpei Tanisawa, Tomio Arai, Seijiro Mori, Motoji Sawabe, Makiko Naka-Mieno, Yoshiji Yamada, Miki Yamada, Noriko Sato, Masaaki Muramatsu, Masashi Tanaka, Yoko Irukayama-Tomobe, Yuki C. Saito, Takeshi Sakurai, Masakazu Hayashida, Haruhiko Sugimura, Kazutaka Ikeda

**Affiliations:** Addictive Substance Project, Tokyo Metropolitan Institute of Medical Science, 2-1-6 Kamikitazawa, Setagaya-ku, Tokyo 156-8506 Japan; Department of Clinical Nursing, Hamamatsu University School of Medicine, Hamamatsu, 431-3192 Japan; Department of Tumor Pathology, Hamamatsu University School of Medicine, Hamamatsu, 431-3192 Japan; Department of Pathology, Iwata City Hospital, Iwata, 438-8550 Japan; Department of Surgery, Toho University Sakura Medical Center, Sakura, 285-8741 Japan; Department of Anesthesiology, Toho University Sakura Medical Center, Sakura, 285-8741 Japan; Ujike Nishiguchi Clinic, Okayama, 700-0024 Japan; Department of Psychiatry, Nagoya University Graduate School of Medicine, Nagoya, 466-8550 Japan; Department of Psychiatry, Seiwa Hospital, Institute of Neuropsychiatry, Tokyo, 162-0851 Japan; Department of Psychiatry, Fujita Health University School of Medicine, Toyoake, 470-1192 Japan; Department of Psychiatry, Kobe University Graduate School of Medicine, Kobe, 650-0017 Japan; Department of Psychiatry, Graduate School of Medicine, Chiba University, Chiba, 260-8670 Japan; Department of Neuropsychopharmacology, National Institute of Mental Health, National Center of Neurology and Psychiatry, Tokyo, 187-8553 Japan; Seimei Hospital, Fuji City, 417-0801 Japan; Koujin Hospital, Nagoya, 463-8530 Japan; Saitama Seishin-iryo Center, Kita-adachi, Saitama 362-0806 Japan; Kanagawa Psychiatric Center, Serigaya Hospital, Kanagawa, 233-0006 Japan; Schizophrenia and Depression Project, Tokyo Metropolitan Institute of Medical Science, Tokyo, 156-8506 Japan; Department of Psychiatry, Osaka University Graduate School of Medicine, Osaka, 565-0871 Japan; National Hospital Organization, Yamato Mental-Medical Center, Nara, 639-1042 Japan; Molecular Research Center for Children’s Mental Development, United Graduate School of Child Development, Osaka University, Kanazawa University, Hamamatsu University, Chiba University, and Fukui University School of Medicine, Osaka, 565-0871 Japan; Department of Genomics for Longevity and Health, Tokyo Metropolitan Institute of Gerontology, Tokyo, 173-0015 Japan; Graduate School of Sport Sciences, Waseda University, Tokyo, 359-1192 Japan; Department of Pathology, Tokyo Metropolitan Geriatric Hospital, Tokyo, 173-0015 Japan; Center for Promotion of Clinical Investigation, Tokyo Metropolitan Geriatric Hospital, Tokyo, 173-0015 Japan; Molecular Pathophysiology, Department of Molecular-genetic Sciences, Division of Biomedical Laboratory Sciences, Graduate School of Health Care Sciences, Tokyo Medical and Dental University, Tokyo, 101-0062 Japan; Department of Medical Informatics, Center for Information, Jichi Medical University, Shimotsuke, 329-0498 Japan; Department of Human Functional Genomics, Life Science Research Center, Mie University, Tsu, 514-8507 Japan; Department of Molecular Epidemiology, Medical Research Institute, Tokyo Medical and Dental University, Tokyo, 101-0062 Japan; Department of Clinical Laboratory, Tokyo Metropolitan Geriatric Hospital, Tokyo, 173-0015 Japan; International Institute of Integrative Sleep Medicine, Ibaraki, 305-8575 Japan; Department of Molecular Neuroscience and Integrative Physiology, Faculty of Medicine, Kanazawa University, Ishikawa, 920-8640 Japan; Department of Anesthesiology and Pain Medicine, Juntendo University School of Medicine, Tokyo, 113-8421 Japan; Japanese Genetics Initiative for Drug Abuse (JGIDA), Tokyo, Japan

## Abstract

**Background:**

Many genetic and environmental factors are involved in the etiology of nicotine dependence. Although several candidate gene variations have been reported by candidate gene studies or genome-wide association studies (GWASs) to be associated with smoking behavior and the vulnerability to nicotine dependence, such studies have been mostly conducted with subjects with European ancestry. However, genetic factors have rarely been investigated for the Japanese population as GWASs. To elucidate genetic factors involved in nicotine dependence in Japanese, the present study comprehensively explored genetic contributors to nicotine dependence by using whole-genome genotyping arrays with more than 200,000 markers in Japanese subjects.

**Results:**

The subjects for the GWAS and replication study were 148 and 374 patients, respectively. A two-stage GWAS was conducted using the Fagerström Test for Nicotine Dependence (FTND), Tobacco Dependence Screener (TDS), and number of cigarettes smoked per day (CPD) as indices of nicotine dependence. For the additional association analyses, patients who underwent major abdominal surgery, patients with methamphetamine dependence/psychosis, and healthy subjects with schizotypal personality trait data were recruited. Autopsy specimens with various diseases were also evaluated. After the study of associations between more than 200,000 marker single-nucleotide polymorphisms (SNPs) and the FTND, TDS, and CPD, the nonsynonymous rs2653349 SNP (located on the gene that encodes orexin [hypocretin] receptor 2) was selected as the most notable SNP associated with FTND, with a *p* value of 0.0005921 in the two-stage GWAS. This possible association was replicated for the remaining 374 samples. This SNP was also associated with postoperative pain, the initiation of methamphetamine use, schizotypal personality traits, and susceptibility to goiter.

**Conclusions:**

Although the *p* value did not reach a conventional genome-wide level of significance in our two-stage GWAS, we obtained significant results in the subsequent analyses that suggest that the rs2653349 SNP (Val308Ile) could be a genetic factor that is related to nicotine dependence and possibly pain, schizotypal personality traits, and goiter in the Japanese population.

**Electronic supplementary material:**

The online version of this article (doi:10.1186/s13041-015-0142-x) contains supplementary material, which is available to authorized users.

## Background

Despite the worldwide spread of cigarette smoking, many tobacco users are reportedly unaware of the harmful chemicals in tobacco products and tobacco smoke and the wide spectrum of specific illnesses that are caused by tobacco use [1]. They also frequently do not know that smoking can cause cancer (other than lung cancer), heart disease, stroke, and many other diseases [2]. Direct tobacco smoking is currently responsible for the death of approximately 5 million people worldwide each year, with many of these deaths occurring prematurely [3, 4]. In Japan, the proportion of deaths that are attributable to tobacco is estimated to be 17 % for ages 30 and over [3]. Therefore, decreasing the frequency of cigarette smoking is imperative to prevent diseases caused by tobacco use. However, many smokers are not easily able to quit smoking because of the addictive property of nicotine, a major component of tobacco.

The heritability of nicotine dependence is estimated to range from 0.40 to 0.75 [5–7], and many genetic and environmental factors have been implicated in the etiology of the disorder. To date, several candidate gene variations have been reported to be associated with smoking behavior and the vulnerability to nicotine dependence. These were found mostly by investigating single-nucleotide polymorphisms (SNPs) on each gene related to specific phenotypes or genome-wide association studies (GWASs), which evaluate genetic markers in the entire genome. By taking the former candidate gene approach, several SNPs have been found to be associated with smoking behavior or nicotine dependence [8, 9]. The latter genome-wide approach has also successfully identified numerous candidate SNPs that may contribute to the etiology of nicotine dependence or smoking-related behavior [10–23]. The strongest signal of association appears to be SNPs at 15q25, represented by a synonymous rs1051730 SNP or nonsynonymous rs16969968 SNP in the region of *CHRNA5*–*CHRNA3*–*CHRNB4*, which encodes neuronal nicotinic acetylcholine receptor subunits [16–21]. However, these studies were conducted with subjects with mostly European or African ancestry [10–23]. Although a recent study that used Korean samples found loci that influence smoking initiation and nicotine dependence other than those identified in studies with samples of European origin [24], the applicability of the results to Japanese or other Asian populations is unknown.

To elucidate the genetic factors that are involved in nicotine dependence in the Japanese population, the present study comprehensively explored genetic contributors to nicotine dependence using whole-genome genotyping arrays with more than 200,000 markers in Japanese subjects.

## Results

### Genome-wide association study identifies a locus associated with nicotine dependence in a Japanese population

In the first exploratory stage analysis of the two-stage GWAS, 11,968, 10,865, and 11,244 SNPs showed *p* values less than the threshold (*p* = 0.05) and were selected as candidates for the second-stage analysis for the FTND, TDS, and CPD. In the second stage analysis and LD-based SNP pruning, 273, 250, and 248 SNPs showed *p* values less than the threshold (*p* = 0.05) for the FTND, TDS, and CPD, respectively. These SNPs were considered potent candidates, and SNPs for the replication study were selected from among these SNPs. The top 50 candidate SNPs with the lowest *p* values for the FTND, TDS, and CPD are presented in Tables 1, 2, and 3, respectively. The top 51–100 candidate SNPs for the FTND, TDS, and CPD are presented in Additional file 1: Table S1, Additional file 2: Table S2, and Additional file 3: Table S3, respectively. Several candidate SNPs after the second-stage analysis were found to be located within or around the gene regions that were already identified as candidate loci associated with nicotine dependence or smoking behavior (Additional file 4: Table S4). Additionally, several SNPs or related genes that were selected after the second-stage analysis commonly appeared in the lists of candidates that resulted from the analysis for different phenotypic measures of nicotine dependence (Additional file 4: Table S4).

**Table 1:** Top 50 candidate SNPs possibly associated with nicotine dependence (FTND score). CHR, chromosome number; Position, chromosomal position (bp); Related gene, the nearest gene from the SNP site

Rank	CHR	SNP	Position	Genotype^b^(FTND ≥ 4)	Genotype^b^(FTND < 4)	χ^*2*^	*p*	Related gene	Region	MAF	TFBS	Splicing	Polyphen
1	6	rs12333016	107205884	0/21/42	0/4/80	20.82	5.04E-06	*QRSL1*	intron	0.08503401	-	-	-
2	2	rs11890985	116384532	2/30/31	0/15/70	19.21	1.17E-05	*DPP10*	3′ flanking	0.16554054	-	-	-
3	13	rs7984684	43517893	0/17/43	8/45/31	19.1	1.24E-05	*LOC121838*	3′ flanking	0.27083333	-	-	-
4	5	rs1009069	167806516	2/22/39	0/9/76	16.41	5.11E-05	*WWC1*	intron	0.11824324	-	-	-
5	5	rs4958493	151090416	0/18/45	0/4/81	16.28	5.45E-05	*ATOX1*	3′ flanking	0.07432432	-	-	-
6	6	rs7754752	54272356	0/7/56	2/33/50	16.12	5.96E-05	*TINAG*	5′ flanking	0.14864865	-	-	-
7	5	rs4976553	167800531	4/33/26	28/39/18	15.56	8.01E-05	*WWC1*	intron	0.45945946	-	-	-
8	13	rs7323018	43513039	3/25/35	16/47/22	15.46	8.42E-05	*LOC121838*	3′ flanking	0.37162162	-	-	-
9	13	rs4631996	98096579	0/3/60	1/25/59	15.06	1.04E-04	*LOC643193*	5′ flanking	0.10135135	-	-	-
10	2	rs10176321	235561801	7/30/26	27/44/14	15	1.08E-04	*SH3BP4*	intron	0.47972973	-	-	-
11	13	rs1768047	98126380	0/6/57	2/30/53	14.99	1.08E-04	*SLC15A1*	3′ flanking	0.13513514	-	-	-
12	4	rs2390944	127169897	0/4/59	1/27/57	14.94	1.11E-04	*LOC645841*	5′ flanking	0.11148649	-	-	-
13	13	rs9533755	43530210	1/20/42	11/42/32	14.54	1.37E-04	*LOC121838*	3′ flanking	0.29054054	-	-	-
14	2	rs2042831	235521853	5/36/22	26/46/13	14.53	1.38E-04	*SH3BP4*	5′ flanking	0.48648649	+	-	-
15	2	rs1542604	131309515	3/22/38	1/9/75	14.47	1.42E-04	*LOC730032*	5′ flanking	0.13175676	+	-	-
16	1	rs188158	26770729	9/33/21	4/26/55	14.44	1.45E-04	*RPS6KA1*	intron	0.28716216	-	-	-
17	13	rs696938	92529022	8/26/29	3/17/65	14.37	1.50E-04	*GPC6*	5′ flanking	0.21959459	-	-	-
18	10	rs7924276	132704843	26/27/10	12/44/29	14.33	1.53E-04	*TCERG1L*	3′ flanking	0.49662162	-	-	-
19	12	rs6488995	124375941	13/32/18	5/32/48	14.24	1.61E-04	*TMEM132B*	5′ flanking	0.33783784	+	-	-
20	13	rs4772116	98100046	0/0/63	0/17/68	14.24	1.61E-04	*LOC643193*	5′ flanking	0.05743243	-	-	-
21	1	rs3895368	228949696	1/13/49	4/42/39	14.13	1.70E-04	*CAPN9*	5′ flanking	0.21959459	+	-	-
22	5	rs2973808	177699186	8/25/30	2/19/64	13.74	2.10E-04	*COL23A1*	intron	0.21621622	-	-	-
23	2	rs4668703	11052055	22/31/10	13/37/35	13.63	2.23E-04	*KCNF1*	3′ flanking	0.46621622	-	-	-
24	15	rs12591960	87909455	3/19/41	11/45/28	13.62	2.24E-04	*C15orf42*	5′ flanking	0.31292517	-	-	-
25	5	rs9314227	97581046	6/31/26	27/41/17	13.46	2.44E-04	*LOC642909*	5′ flanking	0.46621622	-	-	-
26	8	rs3862072	65564638	7/28/28	26/42/17	13.41	2.50E-04	*BHLHB5*	5′ flanking	0.45945946	-	-	-
27	15	rs4887435	84682665	7/26/30	0/24/61	13.23	2.76E-04	*LOC727915*	5′ flanking	0.21621622	-	-	-
28	8	rs7819541	22098096	0/4/59	4/22/59	13.12	2.92E-04	*BMP1*	intron	0.11486486	-	-	-
29	6	rs2021678	102140234	0/5/58	3/25/57	13.12	2.93E-04	*GRIK2*	intron	0.12162162	-	-	-
30	18	rs9303903	2433818	16/38/9	12/35/38	12.83	3.41E-04	*METTL4*	3′ flanking	0.43581081	-	-	-
31	6	rs16882180	52164345	11/35/17	6/31/48	12.83	3.42E-04	*IL17A*	3′ flanking	0.33783784	-	-	-
32	7	rs11771393	154410032	1/4/58	3/27/54	12.79	3.49E-04	*PAXIP1*	intron	0.13265306	-	-	-
33	6	rs17082273	152529619	0/2/61	0/21/64	12.78	3.50E-04	*SYNE1*	intron	0.0777027	-	+	-
34	16	rs11862729	14053599	6/38/19	3/31/51	12.77	3.53E-04	*MKL2*	5′ flanking	0.29391892	-	-	-
35	3	rs6807108	146970672	6/30/27	3/20/62	12.73	3.60E-04	*LOC389156*	3′ flanking	0.22972973	-	-	-
36	10	rs1909669	123376244	18/34/11	9/42/34	12.6	3.87E-04	*LOC729426*	intron	0.43918919	-	-	-
37	13	rs1927382	101940250	1/17/45	0/6/79	12.55	3.97E-04	*FGF14*	5′ flanking	0.08445946	-	-	-
38	2	rs17028222	60529867	1/10/52	3/37/45	12.52	4.03E-04	*BCL11A*	3′ flanking	0.18581081	-	-	-
39	6	rs2237204	16532935	14/31/18	6/33/46	12.51	4.04E-04	*ATXN1*	intron	0.35135135	-	-	-
40	21	rs2242936	39345341	0/8/55	1/32/52	12.44	4.20E-04	*LOC391282*	5′ flanking	0.14189189	-	-	-
41	7	rs715205	122188782	0/14/49	0/3/82	12.44	4.21E-04	*CADPS2*	intron	0.05743243	-	-	-
42	16	rs289741	55574975	7/27/29	19/51/15	12.4	4.29E-04	*CETP*	intron	0.43918919	-	-	-
43	10	rs12416632	37323355	8/24/31	21/47/17	12.2	4.78E-04	*LOC646348*	5′ flanking	0.43581081	+	-	-
44	1	rs7512726	204899584	9/34/20	3/33/49	12.19	4.81E-04	*DYRK3*	3′ flanking	0.30743243	-	-	-
45	14	rs11627505	85145744	0/15/48	9/32/44	12.06	5.16E-04	*FLRT2*	5′ flanking	0.21959459	-	-	-
46	1	rs1891243	222754068	2/28/33	1/16/68	12.01	5.29E-04	*WDR26*	5′ flanking	0.16891892	-	-	-
47	17	rs2309381	5209436	24/29/10	11/48/26	11.99	5.35E-04	*RABEP1*	intron	0.49662162	-	-	-
48	2	rs10166654	236276002	9/25/29	26/42/17	11.97	5.41E-04	*CENTG2*	intron	0.46283784	-	-	-
49	7	rs10230426	18699683	2/27/34	1/15/69	11.89	5.66E-04	*HDAC9*	intron	0.16216216	-	-	-
50	6	rs2653349	55250296	0/15/48	0/4/81	11.8	5.92E-04	*HCRTR2*	non-syn^a^	0.06418919	-	+	benign

^a^, coding region (nonsynonymous polymorphism);

^b^, distribution of genotype (homozygote of minor allele/heterozygote/homozygote of major allele);

MAF, minor allele frequency; TFBS, SNPs located at a transcription factor-binding site (TFBS) of a gene;

Splicing, SNPs located at exonic splicing enhancer (ESE) or exonic splicing silencer (ESS);

Polyphen, predicted possible impact of an amino acid substitution in nonsynonymous SNPs on the structure and function of a human protein by PolyPhen tool (http://genetics.bwh.harvard.edu/pph/)

**Table 2:** Top 50 candidate SNPs possibly associated with nicotine dependence (TDS score)

Rank	CHR	SNP	Position	Genotype^b^(TDS ≥ 3)	Genotype^b^(TDS < 3)	χ^*2*^	*p*	Related gene	Region	MAF	TFBS	Splicing	Polyphen
1	18	rs11662608	60876992	7/38/33	22/36/11	18.28	1.90E-05	*CDH7*	5′ flanking	0.44897959	-	-	-
2	2	rs10490099	58991504	6/40/33	0/19/50	16.33	5.33E-05	*LOC730134*	3′ flanking	0.23986486	-	-	-
3	13	rs17056451	37163539	8/44/24	2/22/45	16.24	5.59E-05	*TRPC4*	intron	0.29655172	-	-	-
4	4	rs13112895	60509294	18/40/21	5/24/40	16.17	5.80E-05	*LOC644419*	5′ flanking	0.37162162	-	-	-
5	9	rs10973120	36885382	5/20/54	9/38/22	15.88	6.76E-05	*PAX5*	intron	0.29054054	-	-	-
6	18	rs611411	60899889	26/43/10	10/31/28	15.82	6.95E-05	*CDH7*	5′ flanking	0.49324324	-	-	-
7	18	rs176136	60936692	18/43/18	5/28/36	15.61	7.80E-05	*CDH7*	5′ flanking	0.39527027	-	-	-
8	18	rs176121	60958616	14/42/23	4/23/42	15.28	9.28E-05	*CDH7*	5′ flanking	0.34121622	-	-	-
9	2	rs985162	129883821	10/44/25	0/29/40	15.18	9.76E-05	*LOC402102*	5′ flanking	0.31418919	-	-	-
10	5	rs1366589	84922599	11/35/33	4/12/53	15.17	9.81E-05	*LOC645181*	3′ flanking	0.26013514	-	-	-
11	18	rs12962611	18303667	5/47/27	0/25/44	15.1	1.02E-04	*CTAGE1*	5′ flanking	0.27702703	-	-	-
12	1	rs909530	169349798	16/46/17	4/31/34	15.06	1.04E-04	*FMO3*	syn^a^	0.39527027	-	+	-
13	1	rs12032382	207272695	0/10/69	0/28/41	15.05	1.05E-04	*LOC391157*	5′ flanking	0.12837838	-	-	-
14	13	rs9532107	37187961	2/24/53	13/28/28	15.01	1.07E-04	*TRPC4*	intron	0.27702703	-	-	-
15	5	rs7719216	8023898	3/20/56	7/36/26	14.97	1.09E-04	*MTRR*	3′ flanking	0.25675676	-	-	-
16	4	rs923665	38214103	0/5/72	1/20/46	14.96	1.10E-04	*NULL*	3′ flanking	0.09375	-	-	-
17	15	rs28716882	21552860	3/28/48	0/8/61	14.94	1.11E-04	*NDN*	5′ flanking	0.14189189	-	-	-
18	2	rs875063	111529556	10/32/37	20/37/12	14.92	1.12E-04	*ACOXL*	intron	0.43581081	-	-	-
19	4	rs13144500	183475934	1/9/69	3/26/40	14.91	1.13E-04	*ODZ3*	intron	0.14527027	-	-	-
20	6	rs1772253	24528213	4/35/40	0/14/55	14.76	1.22E-04	*MRS2L*	intron	0.19256757	-	-	-
21	4	rs2866688	78968066	4/38/37	2/11/55	14.74	1.24E-04	*CNOT6L*	5′ flanking	0.20748299	-	-	-
22	12	rs17120420	56856068	18/44/16	4/34/31	14.45	1.44E-04	*LOC338805*	5′ flanking	0.41496599	-	-	-
23	17	rs16946572	12604902	7/30/42	17/35/17	14.32	1.54E-04	*MYOCD*	intron	0.38175676	-	-	-
24	18	rs4328535	70129056	12/39/28	0/28/41	14.16	1.68E-04	*DKFZP781G0119*	5′ flanking	0.30743243	+	-	-
25	16	rs10514585	81841839	7/43/29	22/35/12	14.16	1.68E-04	*CDH13*	intron	0.45945946	-	-	-
26	16	rs1379652	17304099	13/39/25	2/26/41	14.09	1.74E-04	*XYLT1*	intron	0.32534247	-	-	-
27	6	rs2496167	13450101	9/33/37	17/40/12	13.91	1.92E-04	*TBC1D7*	5′ flanking	0.4222973	-	-	-
28	11	rs623312	31864040	19/43/17	5/32/32	13.76	2.08E-04	*LOC729605*	5′ flanking	0.41554054	-	-	-
29	2	rs1403858	36566018	0/23/55	0/4/65	13.7	2.14E-04	*CRIM1*	intron	0.09183673	-	-	-
30	6	rs1591996	8291461	0/3/76	0/17/52	13.69	2.16E-04	*SLC35B3*	3′ flanking	0.06756757	-	-	-
31	9	rs4877369	89514689	8/26/45	19/30/20	13.65	2.20E-04	*DAPK1*	3′ flanking	0.37162162	-	-	-
32	16	rs17765854	64608419	21/43/15	7/31/31	13.63	2.22E-04	*CDH5*	5′ flanking	0.43918919	-	-	-
33	5	rs540375	152858920	2/23/54	0/5/64	13.59	2.27E-04	*GRIA1*	intron	0.10810811	-	-	-
34	16	rs2305689	69354016	10/30/39	19/36/14	13.48	2.41E-04	*VAC14*	intron	0.41891892	-	-	-
35	8	rs2975701	10147867	0/8/71	1/23/45	13.44	2.47E-04	*MSRA*	intron	0.11148649	-	-	-
36	2	rs1567852	174440084	1/22/56	0/4/65	13.33	2.61E-04	*LOC344178*	3′ flanking	0.09459459	-	-	-
37	7	rs1540893	143398486	18/31/29	2/26/41	13.27	2.70E-04	*OR2A25*	5′ flanking	0.32993197	+	-	-
38	1	rs6686733	4663049	4/28/47	0/10/59	13.26	2.71E-04	*AJAP1*	intron	0.15540541	-	-	-
39	13	rs9555699	109898019	3/28/48	12/33/24	13.12	2.92E-04	*COL4A2*	intron	0.30743243	-	-	-
40	15	rs10851750	63908644	6/26/47	17/29/23	13.03	3.06E-04	*DENND4A*	5′ flanking	0.34121622	-	-	-
41	10	rs520887	78720167	3/29/47	0/10/59	12.98	3.15E-04	*KCNMA1*	intron	0.15202703	-	-	-
42	6	rs573668	13446548	1/30/48	11/32/26	12.94	3.21E-04	*TBC1D7*	5′ flanking	0.29054054	-	-	-
43	20	rs4810359	40624749	10/30/39	1/16/52	12.84	3.39E-04	*PTPRT*	intron	0.22972973	-	-	-
44	12	rs10771391	27966867	23/41/15	6/36/27	12.84	3.40E-04	*PTHLH*	3′ flanking	0.45608108	-	-	-
45	14	rs2415449	37667678	13/41/25	2/28/39	12.83	3.42E-04	*LOC729122*	intron	0.33445946	-	-	-
46	7	rs3807171	37875304	11/39/29	2/23/44	12.82	3.43E-04	*TXNDC3*	intron	0.2972973	-	-	-
47	4	rs7667301	53395322	3/31/45	12/35/22	12.79	3.48E-04	*RASL11B*	5′ flanking	0.32432432	-	-	-
48	17	rs2188889	13178372	3/27/49	10/35/24	12.72	3.62E-04	*HS3ST3A1*	3′ flanking	0.2972973	-	-	-
49	5	rs6875661	54360379	9/37/33	16/43/10	12.65	3.76E-04	*GZMK*	intron	0.43918919	-	-	-
50	7	rs2058024	25742215	15/39/25	3/27/39	12.57	3.92E-04	*LOC646588*	5′ flanking	0.34459459	-	-	-

CHR, chromosome number; Position, chromosomal position (bp); Related gene, the nearest gene from the SNP site;

^a^, coding region (synonymous polymorphism);

^b^, distribution of genotype (homozygote of minor allele/heterozygote/homozygote of major allele);

MAF, minor allele frequency; TFBS, SNPs located at a transcription factor-binding site (TFBS) of a gene;

Splicing, SNPs located at exonic splicing enhancer (ESE) or exonic splicing silencer (ESS);

Polyphen, predicted possible impact of an amino acid substitution in nonsynonymous SNPs on the structure and function of a human protein by PolyPhen tool (http://genetics.bwh.harvard.edu/pph/)

**Table 3:** Top 50 candidate SNPs possibly associated with nicotine dependence (CPD)

Rank	CHR	SNP	Position	Genotype^b^(CPD > 20)	Genotype^b^(CPD ≤ 20)	χ^*2*^	*p*	Related gene	Region	MAF	TFBS	Splicing	Polyphen
1	2	rs726016	234390241	0/21/15	0/20/92	22.29	2.35E-06	*LOC339766*	non-syn^a^	0.13851351	-	+	probablydamaging
2	2	rs960615	151441447	4/20/12	3/25/84	20.45	6.11E-06	*LOC727840*	3′ flanking	0.19932432	-	-	-
3	2	rs11683427	127673013	1/10/25	0/5/107	19.71	9.00E-06	*CYP27C1*	intron	0.05743243	-	-	-
4	17	rs4077548	75345351	8/25/3	10/44/57	19.14	1.21E-05	*ENPP7*	3′ flanking	0.35714286	-	-	-
5	7	rs2888674	150141848	3/22/11	1/34/77	18.94	1.35E-05	*TMEM176A*	3′ flanking	0.21621622	-	-	-
6	7	rs4721629	17542242	1/17/18	0/18/94	18.26	1.93E-05	*AHR*	3′ flanking	0.125	-	-	-
7	15	rs10518761	54535156	0/9/27	0/3/109	18.22	1.97E-05	*UNC13C*	intron	0.04054054	-	-	-
8	18	rs8093458	65163508	7/20/9	2/47/62	17.45	2.94E-05	*DOK6*	5′ flanking	0.28911565	-	-	-
9	21	rs8127336	27297806	3/12/21	0/15/97	17.08	3.59E-05	*ADAMTS5*	5′ flanking	0.11148649	-	-	-
10	10	rs10904919	17374751	0/18/18	0/18/94	17.04	3.67E-05	*ST8SIA6*	3′ flanking	0.12162162	-	-	-
11	7	rs12540772	150160296	6/20/10	3/40/69	16.8	4.16E-05	*ABP1*	5′ flanking	0.26351351	-	-	-
12	2	rs1542604	131309515	3/14/19	1/17/94	16.35	5.26E-05	*LOC730032*	5′ flanking	0.13175676	+	-	-
13	1	rs10494797	197762985	6/16/14	2/33/77	15.39	8.75E-05	*LOC647202*	3′ flanking	0.21959459	-	-	-
14	1	rs6683501	197753037	6/15/15	0/36/76	15.37	8.84E-05	*LOC647202*	3′ flanking	0.21283784	-	-	-
15	20	rs4811238	36491682	0/0/36	1/36/75	15.35	8.95E-05	*CTNNBL1*	intron	0.12837838	-	-	-
16	7	rs11772167	150134012	2/13/21	0/16/96	14.76	1.22E-04	*TMEM176A*	3′ flanking	0.11148649	+	-	-
17	21	rs8126747	41902438	7/18/11	5/37/70	14.59	1.33E-04	*TMPRSS2*	5′ flanking	0.26689189	-	-	-
18	15	rs1434462	77306926	0/8/28	11/54/47	14.4	1.48E-04	*LOC646956*	5′ flanking	0.28378378	-	-	-
19	7	rs9792016	13823760	7/19/10	7/35/70	14.34	1.53E-04	*LOC646161*	5′ flanking	0.27702703	-	-	-
20	21	rs2830596	27276913	7/19/10	5/41/66	14.22	1.62E-04	*ADAMTS5*	5′ flanking	0.28378378	-	-	-
21	2	rs11902906	143299978	3/13/20	26/63/23	14.21	1.64E-04	*KYNU*	5′ flanking	0.4527027	-	-	-
22	1	rs6658450	197694872	6/17/13	2/38/72	14.12	1.72E-04	*LOC647202*	3′ flanking	0.23986486	-	-	-
23	1	rs1416127	56265694	1/20/15	0/29/83	14.12	1.72E-04	*LOC127406*	3′ flanking	0.1722973	-	-	-
24	14	rs799045	81687929	5/9/22	33/54/25	14.11	1.72E-04	*LOC730103*	5′ flanking	0.46959459	-	-	-
25	7	rs3814991	18601428	10/21/5	12/48/52	14.03	1.80E-04	*HDAC9*	intron	0.38175676	-	-	-
26	4	rs7659741	130342701	10/18/8	6/54/52	13.93	1.90E-04	*LOC391697*	5′ flanking	0.35135135	-	-	-
27	10	rs1570937	18798899	0/11/24	0/8/103	13.79	2.04E-04	*CACNB2*	intron	0.06506849	-	-	-
28	4	rs13435610	181024788	2/17/17	0/26/86	13.79	2.05E-04	*LOC643179*	3′ flanking	0.15878378	-	-	-
29	12	rs11613339	39239162	1/16/19	0/20/92	13.77	2.06E-04	*NULL*	intron	0.12837838	-	-	-
30	6	rs4305747	132959305	0/17/19	21/63/28	13.76	2.08E-04	*TAAR5*	5′ flanking	0.41216216	+	-	-
31	8	rs7006843	117669783	0/7/29	10/51/51	13.69	2.16E-04	*EIF3H*	3′ flanking	0.26351351	-	-	-
32	11	rs3107636	122287366	4/18/14	4/26/82	13.68	2.17E-04	*C11orf63*	intron	0.2027027	-	-	-
33	3	rs13085035	30763983	3/18/15	1/30/81	13.67	2.18E-04	*GADL1*	intron	0.18918919	-	-	-
34	2	rs2698537	64363458	1/12/23	0/12/100	13.66	2.19E-04	*LOC130773*	3′ flanking	0.08783784	-	-	-
35	19	rs4926219	14441328	2/11/23	0/13/99	13.64	2.21E-04	*PKN1*	non-syn^a^	0.09459459	-	+	benign
36	3	rs1157438	22343099	9/14/13	3/43/66	13.54	2.33E-04	*LOC728516*	3′ flanking	0.27364865	-	-	-
37	7	rs6980179	150156343	9/20/7	8/50/54	13.5	2.38E-04	*TMEM176A*	3′ flanking	0.35135135	-	-	-
38	1	rs10518473	74924774	1/9/26	0/7/105	13.37	2.55E-04	*C1orf173*	5′ flanking	0.06081081	-	-	-
39	4	rs4616770	130292102	11/13/12	4/49/59	13.35	2.58E-04	*LOC391697*	5′ flanking	0.31081081	-	-	-
40	3	rs2337708	18169752	0/11/25	0/8/104	13.35	2.59E-04	*SATB1*	3′ flanking	0.06418919	-	-	-
41	1	rs17461925	201293214	5/14/17	1/29/82	13.16	2.86E-04	*PPFIA4*	intron	0.18581081	-	-	-
42	2	rs4851890	98164176	6/18/12	3/40/69	13.1	2.95E-04	*VWA3B*	intron	0.25675676	-	-	-
43	2	rs6759709	2026404	0/2/34	5/38/69	13.01	3.09E-04	*MYT1L*	intron	0.16891892	-	-	-
44	18	rs1567041	4485320	16/12/8	14/52/46	12.99	3.14E-04	*LOC388458*	5′ flanking	0.41891892	-	-	-
45	3	rs149863	138965053	4/12/20	0/23/89	12.84	3.39E-04	*SOX14*	5′ flanking	0.14527027	+	-	-
46	11	rs7945313	91134747	12/12/12	5/50/57	12.83	3.41E-04	*LOC727998*	3′ flanking	0.32432432	-	-	-
47	19	rs10417021	62642189	0/12/24	0/10/102	12.82	3.43E-04	*ZNF749*	5′ flanking	0.07432432	-	-	-
48	25	rs5988531	884198	2/21/13	2/32/78	12.79	3.48E-04	*SHOX* | *CRLF2*	intergenic	0.20608108	not available	not available	not available
49	4	rs17051378	122785148	8/17/11	4/46/62	12.72	3.62E-04	*ANXA5*	3′ flanking	0.29391892	-	-	-
50	11	rs4756986	19056814	7/15/14	4/34/74	12.68	3.69E-04	*MRGPRX2*	5′ flanking	0.23986486	-	-	-

CHR, chromosome number; Position, chromosomal position (bp); Related gene, the nearest gene from the SNP site;

^a^, coding region (nonsynonymous polymorphism);

^b^, distribution of genotype (homozygote of minor allele/heterozygote/homozygote of major allele);

MAF, minor allele frequency; TFBS, SNPs located at a transcription factor-binding site (TFBS) of a gene;

Splicing, SNPs located at exonic splicing enhancer (ESE) or exonic splicing silencer (ESS);

Polyphen, predicted possible impact of an amino acid substitution in nonsynonymous SNPs on the structure and function of a human protein by PolyPhen tool (http://genetics.bwh.harvard.edu/pph/)

Although the *p* values for the top candidate SNPs were not sufficiently low when considering the conventional genome-wide level of significance, the list of the top 50 candidate SNPs (Tables 1, 2, 3) was found to include a few nonsynonymous SNPs that involve amino-acid changes and are expected to have a greater impact on the functions of gene products than other SNPs. Therefore, the best of these candidates for the FTND and CPD, rs2653349 (Rank 50) and rs726016 (Rank 1), respectively, were considered for the further confirmatory replication study (Tables 1 and 3). In the replication study, of the two SNPs, only the rs2653349 SNP showed a significant association with FTND score (χ^2^ = 5.094, *q* = 0.048; Table 4). This SNP was within the region of the *HCRTR2* gene, which encodes orexin (hypocretin) receptor 2.

**Table 4:** Results of confirmatory association analysis between the selected SNPs and nicotine dependence

SNP	CHR	Position	Phenotype	Exploratory		Confirmatory			Combined		Genotype^a^(FTND ≥ 4)(CPD > 20)	Genotype^a^(FTND < 4)(CPD ≤ 20)	Related gene
				χ^*2*^	*p*	χ^*2*^	*p*	*q*	χ^*2*^	*p*			
rs2653349	6	55250296	FTND	12.3	0.0005921	5.094	0.024	0.048*	14.02	0.0001806	0/41/203	0/17/252	*HCRTR2*
rs726016	2	234390241	CPD	22.29	0.00000235	0.471	0.4925	0.4925	3.023	0.08209	2/41/94	4/87/293	*LOC339766*

CHR, chromosome number; Position, chromosomal position (bp); ^a^, distribution of genotype (homozygote of minor allele/heterozygote/homozygote of major allele); *, corrected *p* < 0.05

**Fig. 1:**
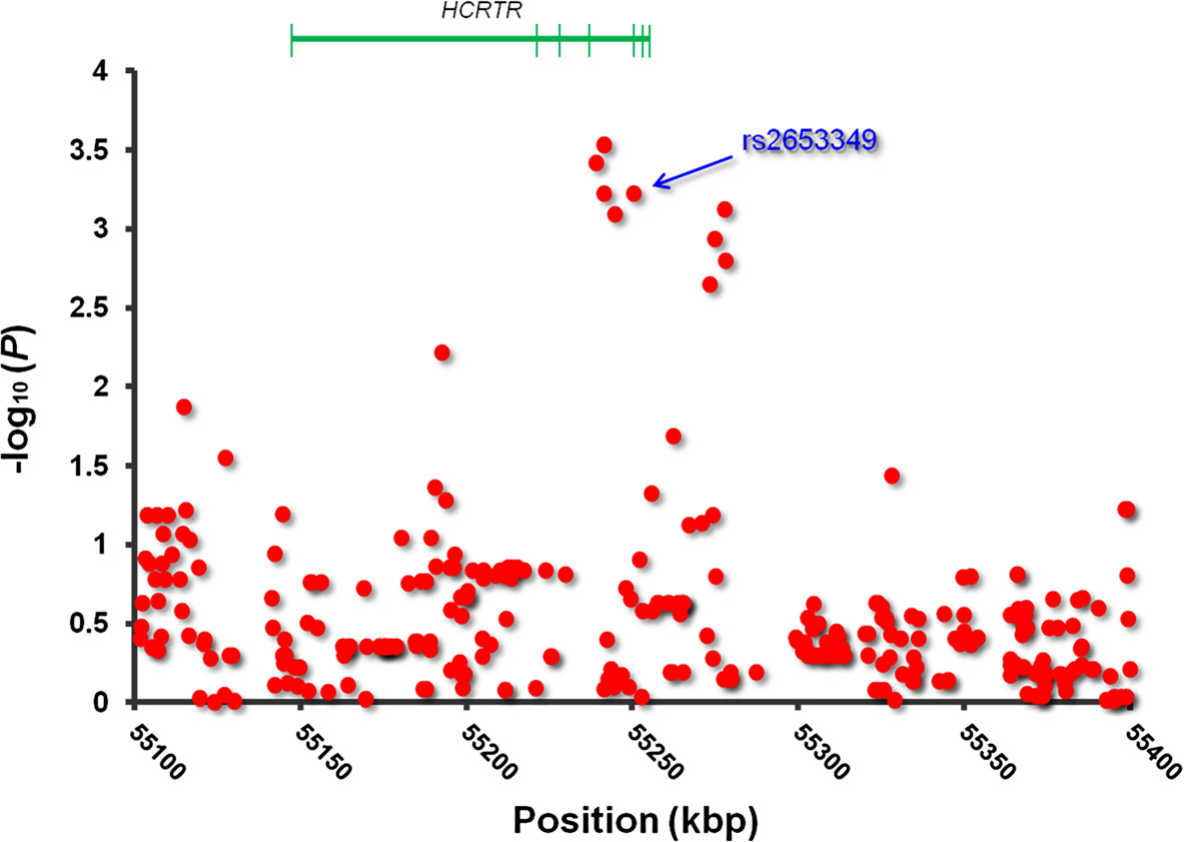
Fine mapping of the candidate region after the imputation-based association analysis. The circle plot represents the results of the association analysis in a trend model using the GWAS samples. The range for the X-axis represents the genomic position from 55,100,000 to 55,400,000 on chromosome 6 in the HapMap database (http://hapmap.ncbi.nlm.nih.gov/index.html.ja; accessed July 15, 2015). The green object above the plot illustrates the schematic structure of the human *HCRTR* gene (Gene Accession no. NM_001526.3), based on the NCBI database (http://www.ncbi.nlm.nih.gov/gene/; accessed July 15, 2015). The gene is illustrated between the 5’- and 3’-flanking regions, corresponding to left and right sides, respectively. The green vertical lines and horizontal line that connects them represent the exon and intron, respectively

Imputation analysis was further conducted for SNPs around the rs2653349 SNP for the GWAS samples. As a result, several SNPs whose genotypes were imputed exhibited strong associations similarly to the rs2653349 SNP. These SNPs (rs2653342, rs2653343, rs2653344, and rs2653347) were located at a slightly upstream region of the rs2653349 SNP (Fig. 1). However, our database search with SNPinfo Web Server (see Additional file 5: Supporting Information) revealed that all of these SNPs were in absolute linkage disequilibrium with the rs2653349 SNP (*D*′ = *r*^2^ = 1). Given that genotype call ratios by whole-genome genotyping or imputation for the rs2653342, rs2653343, rs2653344, rs2653347, and rs2653349 SNPs were 99.0 %, 97.7 %, 100.0 %, 98.0 %, 100.0 %, and 100.0 %, respectively, the apparently stronger associations that were observed for the rs2653342, rs2653343, and rs2653347 SNPs than for the rs2653349 SNP were merely attributable to the incomplete genotype data for these SNPs. Therefore, the contribution of the rs2653342, rs2653343, rs2653344, and rs2653347 SNPs to nicotine dependence was regarded as statistically equal to that of the rs2653349 SNP. Considering that rs2653349 is a nonsynonymous SNP and the others are located in the intronic region of the *HCRTR2* gene, one could assume that this SNP is most likely the causative SNP.

### Association between rs2653349 SNP and postoperative pain in patients who underwent major open abdominal surgery

To examine whether the rs2653349 SNP that was identified in our GWAS affects individual differences in the sensitivity to some drugs and pain, we investigated the association between the rs2653349 SNP and postoperative opioid requirements and pain in another cohort who underwent major open abdominal surgery (Additional file 6: Table S5). The subjects included 112 patients who underwent major open abdominal surgery under combined general and epidural anesthesia (Additional file 6: Table S5). Significant associations were not found for the frequency of analgesic administration (*U* = 495, *p* = 0.1373; Additional file 6: Table S5) or the total dose administered (*U* = 480, *p* = 0.1091; Additional file 6: Table S5). However, a significant difference in pain intensity at rest during the first 24-h postoperative period, reflected by scores on a numerical rating scale (NRS; i.e., a 5-point verbal rating scale, in which 0 = no pain, 1 = mild pain, 2 = moderate pain, 3 = severe pain, and 4 = extremely severe pain), was found between subjects with the A/G genotype and subjects with the G/G genotype in the rs2653349 SNP. Furthermore, carriers of the G/G genotype complained more about pain than carriers of the A/G genotype (*U* = 212, *p* = 0.0017; Additional file 6: Table S5), suggesting that G/G carriers are more sensitive to postoperative pain than A/G carriers.

### Association between rs2653349 SNP and the initiation of drug use in patients with METH dependence

We also investigated the association between the rs2653349 SNP and two measures of the severity of METH dependence: age at first use of the drug (years) and number of drugs used, including METH. Significant associations were not found for the number of drugs used (*U* = 1894, *p* = 0.2601; Additional file 7: Table S6). However, a significant difference was found in the first use of METH between the subjects with the A/G genotype and subjects with the G/G genotype in the rs2653349 SNP. Carriers of the A/G genotype first used METH at a significantly younger age than carriers of the G/G genotype (*U* = 1097, *p* = 0.0056; Additional file 7: Table S6), suggesting that A/G carriers might harbor a high risk of initiating METH use and becoming regular users at younger ages than G/G carriers.

### Association between rs2653349 SNP and schizotypal personality trait in healthy subjects

To further examine the impact of the rs2653349 SNP on individual differences in the vulnerability to develop other psychiatric diseases in healthy subjects, we examined the possible effect of the rs2653349 genotype in the *HCRTR2* gene on total Schizotypal Personality Questionnaire (SPQ) scores [25–27] using a one-way analysis of covariance (ANCOVA). To control confounding factors, age, sex, and years of education were used as covariates [28]. This analysis indicated a significant effect of genotype (*F*_1,306_ = 5.80, *p* = 0.017; Additional file 8: Table S7). We then investigated the effect of genotype on the three SPQ factors: cognitive/perceptual, interpersonal, and disorganization [29]. Significant effects of genotype were found on the cognitive/perceptual factor (*F*_1,306_ = 4.37, *p* = 0.037) and disorganization factor (*F*_1,306_ = 4.19, *p* = 0.042; Additional file 9: Table S8), but no effect of genotype was found on the interpersonal factor (*p* > 0.09). Subjects with the risk A/G genotype of the rs2653349 SNP had higher schizotypal trait scores than subjects with the G/G genotype (Additional file 9: Table S8).

### Association between rs2653349 SNP and goiter in samples of autopsy specimens

We also investigated whether the rs2653349 SNP affects the susceptibility to diseases other than psychiatric diseases in another cohort by evaluating the association between this SNP and a total of 104 diseases/phenotypes using autopsy specimens (Additional file 10: Table S9). Nominally significant associations were found between goiter, aortic aneurysm, myeloma, and amyotrophic lateral sclerosis and the rs2653349 SNP. Carriers of the A/G genotype were more frequently in the disease group than in the control group (Additional file 11: Table S10). The association between this SNP and goiter was much stronger than the associations with the other three diseases (Additional file 11: Table S10). These results suggest that A/G carriers may harbor a higher risk of developing these diseases than G/G carriers.

## Discussion

Previous GWASs on nicotine dependence that used samples of mainly European origin identified numerous candidate SNPs [10–23]. Several of the candidate SNPs in the present study after the second-stage analysis were found to be located within or around the gene regions that have already been identified as candidate loci associated with nicotine dependence or smoking behavior (Additional file 4: Table S4). These candidate genetic factors may be common to some populations other than Japanese. Additionally, several SNPs or related genes that were selected after the second-stage analysis commonly appeared in the lists of candidates that resulted from the analysis of different phenotypic measures of nicotine dependence (Additional file 4: Table S4). These SNPs/genes might be candidates that possibly contribute to several aspects of dependence simultaneously. Among these candidate SNPs/genes were *CSMD1* and *IMMP2L*, which were selected as candidate genes to test associations with the FTND, TDS, and CPD (Additional file 4: Table S4). *CSMD1*, a tumor suppressor gene [30], is a known candidate for nicotine dependence [11]. The SNPs in the region of *CHRNA5*–*CHRNA3*–*CHRNB4* at 15q25, which have been reported to show strong associations with CPD in studies with subjects with European ancestry [16–18], were not included in our list of the top 100 candidates (Tables 1, 2, 3, Additional file 1: Table S1, Additional file 2: Table S2, Additional file 3: Table S3). Although definitive conclusions cannot be drawn until further studies are conducted with larger sample sizes, the SNPs in this region might not greatly affect the severity of nicotine dependence in the Japanese population.

The best candidate SNP, rs2653349, is located within the *HCRTR2* gene on chromosome 6 (Table 4). The *HCRTR2* gene encodes orexin receptor 2. The orexin receptor has two subtypes (orexin receptor 1 [HCRTR1] and orexin receptor 2 [HCRTR2]) and two known neuropeptide ligands (orexin A, which binds both HCRTR1 and HCRTR2, and orexin B, which binds only HCRTR2). Orexin peptides, whose mRNA initially identified them as a hypothalamus-specific peptides with neuroexcitatory activity [31], and their receptors are distributed in various tissues, including the hypothalamus and pituitary [32–34]. They are well known to regulate feeding behavior and states of sleep/wakefulness [32, 35]. Orexin knockout mice were reported to exhibit an attenuation of morphine dependence [36]. The hypocretin receptor 1 antagonist SB334867 and hypocretin receptor 1/2 antagonist almorexant both dose-dependently reduced nicotine self-administration [37], suggesting the involvement of the orexin system in the rewarding effects.

Our further study that examined whether the rs2653349 SNP affects individual differences in the susceptibility to other diseases or phenotypic traits suggested that A/G genotype carriers of this SNP are less sensitive to postoperative pain (Additional file 6: Table S5), have a high risk of initiating METH use regularly at younger ages (Additional file 7: Table S6), have higher schizotypal trait scores (Additional file 9: Table S8), and harbor a higher risk of developing goiter, aortic aneurysm, and myeloma than G/G carriers (Additional file 11: Table S10). For this SNP [38], several previous reports demonstrated an association with cluster headache [39–41]. Intriguingly, carriers of the G/G genotype have been reported to have a higher disease risk than the other genotypes. Given the modulation of antinociceptive effects by the orexin system in various animal models of pain [42], one speculation is that G/G carriers are generally more sensitive to pain because of dampened activity of the orexin system. The severer levels of nicotine dependence in A/G genotype carriers of this SNP may be attributable to increased rewarding effects related to the orexin system, possibly leading to a higher risk of initiating METH use regularly at younger ages. The higher schizotypal trait scores in A/G carriers than in G/G carriers in the present study may be explained by orexin-induced activation of the mesolimbic dopamine system, which may underlie some of its actions in schizophrenia [43]. Previous reports indicated that nicotine dependence was more frequent in smokers with schizophrenia compared with the general population [44]. Chronic schizophrenia patients also have lower visual analogue scale postoperative pain scores than control patients [45], which appears to be consistent with the trend observed in the present study. Less understood is the involvement of the orexin system in goiter, aortic aneurysm, myeloma, and amyotrophic lateral sclerosis. However, the increased frequency of cigarette smoking associated with severer levels of nicotine dependence in A/G carriers of this SNP may cause an elevation of thiocyanate synthesis [46], which would then enhance the risk of goiter [47–49].

Our database search estimated that the possible impact of the amino-acid substitution from valine to isoleucine by the base substitution from G to A in the rs2653349 SNP on the structure and function of human protein is predicted to be “benign.” Indeed, few studies have investigated the functional alterations caused by the rs2653349 SNP. A previous report only showed that the SNP, located in the exon 5 region, had no effect on orexin binding sites, but the corresponding residue Val308Ile might be at the dimer interface and influence the dimerization process of the receptor [50]. Further functional studies will be needed to provide a clearer explanation for the associations observed in human studies between the SNP and phenotypic traits.

With regard to our initial GWAS that was conducted as a consecutive two-stage analysis, the lowest exploratory *p* value for the entire sample was > ~1 × 10^−6^ (Tables 1, 2, 3), which would have been deemed genome-wide nonsignificant if only a single-stage analysis was performed to calculate conventional Bonferroni- or FDR-corrected *p* values for the total samples to determine statistical significance. The situation would not have been improved even if only non-synonymous SNPs had been considered in the analysis as in previous studies [51, 52] because a total of 1,413 non-synonymous SNPs were included among the 225,591 SNPs that were used in our two-stage GWAS, and the conventionally corrected *p* value of the rs2653349 SNP would have exceeded 0.05. However, “significant” results that are obtained as conventionally corrected *p* values may not always represent true associations, meaning that the results may not be necessarily replicated in other studies, and vice versa. For example, data from the National Human Genome Research Institute GWAS catalog (as of January 31, 2009) showed 1,321 entries of discovered associations with *p* values < 10^−5^, but only 550 of these entries had *p* values < 5 × 10^−8^ [53], which is known as a conventionally corrected conservative threshold for declaring a significant association in a GWAS [54, 55]. In both cases, truly potent candidate SNPs may be included in the outcome of the studies. Furthermore, inconsistencies between genome-wide significance and truly meaningful “significance” have been exemplified by numerous studies. For example, Bierut et al. [19] identified SNPs in the β3 nicotinic cholinergic receptor gene as candidates that contribute to the development of nicotine dependence based on *p* values that were genome-wide nonsignificant. Thorgeirsson et al. [18] subsequently performed a genome-wide association meta-analysis using independent samples and found that SNPs in this gene region were genome-wide significantly associated with CPD (*p* < 5 × 10^−8^). Altogether, these reports suggest that conventionally corrected *p* values for combined samples may not be the sole criteria for identifying true associations between SNPs and specific phenotypes. Nevertheless, we did not obtain results with markedly low *p* values in the present GWAS. Future studies are required to draw more definitive conclusions about associations between the rs2653349 SNP and nicotine dependence and the other traits that were examined in the present study.

## Conclusions

Although the *p* value did not reach a conventional genome-wide level of significance in our two-stage GWAS, we obtained results in the subsequent analyses that suggest that carriers of the A/G genotype of the nonsynonymous rs2653349 SNP are more likely to exhibit more severe levels of nicotine dependence than carriers of the G/G genotype in the Japanese population. This result suggests that this SNP may be a genetic factor that is associated with the exacerbation of nicotine dependence and may serve as a marker that predicts the severity of nicotine dependence in the Japanese population.

## Methods

### Subjects

The participants in the present study were a total of 999 patients who were ambulatory, able to communicate orally, and 60 years of age or older. Of these 999 patients, 148 were included in the two-stage GWAS to explore the association between polymorphisms in the entire human genome and the severity of nicotine dependence. Numerous participants in this study had various smoking habits and completed a questionnaire that contained questions about lifestyle, including alcohol consumption, smoking, diet, and cancer history [56, 57]. The detailed demographic and clinical characteristics of the subjects are provided in Additional file 12: Table S11.

The subsequent study that investigated the associations between the candidate SNPs and sensitivity to pain and analgesia included 112 patient subjects who underwent major open abdominal surgery (age, 28–80 years; 60 males and 52 females), mostly gastrectomy for gastric cancer and colectomy for colorectal cancer [58, 59]. Additionally, 203 patients with methamphetamine (METH) dependence (age, 18–69 years; 165 males and 38 females) who were recruited for a collaborative study of the Japanese Genetics Initiative for Drug Abuse (JGIDA) [60, 61] were also enrolled in the present study. Healthy subjects (age, 18–66 years; 129 males and 182 females) were also recruited for the association study between candidate SNPs and schizotypal personality traits. To investigate the impact of the candidate SNPs on the susceptibility to various diseases, samples of 2,305 consecutive Japanese autopsy cases (1,266 male and 1,039 female; mean age at death, 80.6 ± 8.8 years) who were registered in the Internet database of Japanese SNPs for geriatric research [62] were also used. The demographic and clinical data of all of these subjects are described in the Supporting Information (Additional file 6: Table S5, Additional file 7: Table S6, Additional file 8: Table S7, Additional file 10: Table S9) and previous reports [58–65].

### Smoking behavior data

The results of the questionnaire, especially questions related to smoking, were used in the analysis of the severity of nicotine dependence. Some of them included the Fagerstrӧm Test for Nicotine Dependence (FTND; a test that yields a continuous measure of nicotine dependence) [66] and Tobacco Dependence Screener (TDS; a screening questionnaire for tobacco/nicotine dependence according to the *International Statistical Classification of Diseases and Related Health Problems*, 10th revision [ICD-10], *Diagnostic and Statistical Manual of Mental Disorders*, 3rd edition, revised [DSM-III-R], and DSM-IV), which consists of 10 questions [67]. The questionnaire also included questions about the number of cigarettes smoked per day (CPD) and other smoking-related traits (see Additional file 5: Supporting Information). In the present study, the FTND, TDS, and CPD were used as measures of nicotine dependence (Additional file 12: Table S11).

### Genotyping

#### Whole-genome genotyping and quality control

A total of 300 DNA samples from the patients with smoking behavior data who visited Iwata City Hospital were used for genotyping. Whole-genome genotyping was performed using the Infinium assay II (Illumina, San Diego, CA, USA) with HumanCytoSNP-12 v2.0 (total markers: 301,232) BeadChips (see Additional file 5: Supporting Information).

A total of 225,602 SNP markers survived the initial filtration process as described in the Additional file 5: Supporting Information, and a total of 225,591 SNP markers were finally used for the GWAS after filtration using the Hardy-Weinberg equilibrium test (Additional file 13: Figure S1).

#### Genotyping for specific candidate SNPs

The TaqMan allelic discrimination assay (Life Technologies, Carlsbad, CA, USA) was adopted for a total of 112 and 203 DNA samples from patients who underwent major abdominal surgery and patients with METH dependence/psychosis, respectively. Additional file 14: Table S12 provides the genotype distribution of the candidate SNPs. The details of genomic DNA extraction and the TaqMan allelic discrimination assay are described in the Additional file 5: Supporting Information and previous reports [68].

#### Array-based genotyping procedure for data extraction of a specific SNP

To genotype the rs2653349 SNP for a total of 311 and 2,305 DNA samples from healthy subjects with schizotypal personality trait data and autopsy specimens, respectively, array-based genotyping was conducted. The genotype data for this SNP were extracted from the overall results. In the former case, genotyping was performed using Affymetrix Genome-Wide Human SNP Array 6.0 (Affymetrix, Santa Clara, CA, USA) as previously described [69]. In the latter case, all of the samples were genotyped using Illumina Infinium HumanExome Beadchips (Illumina, CA, USA, USA) with iScan. The details are described in the Additional file 5: Supporting Information.

### Statistical analysis

#### Genome-wide association study

A two-stage GWAS was conducted for patients with smoking behavior data to initially investigate the association between genetic variations and the severity of nicotine dependence as an exploratory study to narrow down candidate SNPs. Among the 300 subjects genotyped, approximately half lacked smoking behavior data that were related to nicotine dependence. Therefore, a total of 148 subjects were used for our two-stage GWAS (68 and 80 subjects for the first- and second-stage analyses, respectively). In our two-stage GWAS, 225,591 SNPs were selected for the analyses.

The FTND, TDS, and CPD data were used as an index of nicotine dependence for the GWAS. Prior to the analyses, the subjects were divided into two subgroups: low dependence (FTND < 4, TDS < 3, CPD ≤ 20) and high dependence (FTND ≥ 4, TDS ≥ 3, CPD > 20). The association with SNPs was explored using Cochran-Armitage trend tests. All of the statistical analyses were performed using gPLINK v. 2.050, PLINK v. 1.07 (http://pngu.mgh.harvard.edu/purcell/plink/; accessed April 19, 2014) [70] and Haploview v. 4.1 [71].

The GWAS procedure is summarized in Additional file 13: Figure S1. In the first-stage analysis with 68 samples, the SNPs that showed *p* < 0.05 were selected as candidate SNPs for the second-stage analysis among the 225,591 SNPs. For these SNPs, the second-stage analysis was conducted with 80 samples, and the SNPs that showed *p* < 0.05 for the single analysis of this stage and combined analysis of the first and second stages were considered potent candidates. The SNPs for further replication analysis were selected from these SNPs after linkage disequilibrium (LD)-based SNP pruning to remove redundant SNPs due to strong LD with each other (see Additional file 5: Supporting Information). In the confirmatory replication study to corroborate the possible association between the SNPs that were selected after the second stage and phenotypic trait, the *q* values of the false discovery rate were calculated to correct for multiple testing, in addition to *p* values based on previous reports [72, 73]. The SNPs that showed *q* < 0.05 in the analysis were considered statistically significant. A log quantile-quantile (QQ) *p*-value plot based on the results of the GWAS for the combined samples was subsequently drawn to check the pattern of the generated *p*-value distribution (Additional file 15: Figure S2).

### Association study for candidate SNP

For all of the genotype data used in the additional analyses after the GWAS, the distributions were checked using the χ^*2*^ test, and the absence of significant deviation from the theoretical distribution expected from Hardy-Weinberg equilibrium was confirmed (Additional file 14: Table S12). To explore the association between the rs2653349 SNP and phenotypes, the Mann–Whitney *U*-test, the χ^*2*^ test, and Fisher’s exact test were performed. The statistical methods and software used in the analyses for the candidate SNP are provided in the Additional file 5: Supporting Information. The criterion for significance was set at *p* < 0.05.

### Additional *in silico* analysis

The databases, power analysis, and imputation analysis, which were based on previous reports [74–76], are described in the Additional file 5: Supporting Information.

## Additional files

Additional file 1: Table S1. Top 51–100 candidate SNPs possibly associated with nicotine dependence (FTND score). (DOC 131 kb) Additional file 2: Table S2. Top 51–100 candidate SNPs possibly associated with nicotine dependence (TDS score). (DOC 127 kb) Additional file 3: Table S3. Top 51–100 candidate SNPs possibly associated with nicotine dependence (CPD). (DOC 126 kb) Additional file 4: Table S4. SNPs or genes commonly included in the GWAS candidates for different phenotypes. (DOC 174 kb) Additional file 5:
**Supporting information (Materials and Methods).** (DOC 124 kb) Additional file 6: Table S5. Demographic and clinical data of the subjects who underwent major abdominal surgery. (DOC 74 kb) Additional file 7: Table S6. Demographic and clinical data of patient subjects with methamphetamine dependence/psychosis. (DOC 72 kb) Additional file 8: Table S7. Demographic variables for subjects included in the SPQ analysis (mean ± SD). (DOC 52 kb) Additional file 9: Table S8. Impact of the *HCRTR2* gene risk variant on schizotypal personality traits (mean ± SD). (DOC 54 kb) Additional file 10: Table S9. Demographic and clinical data of patient subjects with autopsy specimens. (DOC 139 kb) Additional file 11: Table S10. Results of analysis of associations between the rs2653349 SNP and diseases/phenotypes. (DOC 221 kb) Additional file 12: Table S11. Demographic data of patient subjects with clinical data related to smoking behavior. (DOC 77 kb) Additional file 13: Figure S1. Schematic illustration of the two-stage GWAS. Candidate SNPs associated with nicotine dependence in Japanese were selected. (TIFF 4809 kb) Additional file 14: Table S12. Distribution of genotypes of the rs2653349 and rs726016 SNPs and results of tests for Hardy-Weinberg equilibrium. (DOC 69 kb) Additional file 15: Figure S2. Log quantile-quantile (QQ) p-value plot as a result of the genome-wide association study for the combined samples. (a) Plot of the results from the FTND. (b) Plot of the results from the TDS. (c) Plot of the results from the CPD. (TIFF 411 kb)
