# Supplementary material for: Associations between the orexin (hypocretin) receptor 2 gene polymorphism Val308Ile and nicotine dependence in genome-wide and subsequent association studies
Source: Mol Brain. 2015 Aug 20;8:50. doi: 10.1186/s13041-015-0142-x (PMC4546081; doi:10.1186/s13041-015-0142-x)
Supplement: Additional file 1: Table S1. — Top 51–100 candidate SNPs possibly associated with nicotine dependence (FTND score). (DOC 131 kb) [file 13041_2015_142_MOESM1_ESM.doc]

| **Table S1. Top 51-100 candidate SNPs possibly associated with nicotine dependence (FTND score).** | | | | | | | | | | |
| --- | --- | --- | --- | --- | --- | --- | --- | --- | --- | --- |
|  |  |  |  |  |  |  |  |  |  |  |
| **Rank** | **CHR** | **SNP** | **Position** | **Genotype^§^ (FTND ≥ 4)** | **Genotype^§^ (FTND < 4)** | ***χ^2^*** | ***p*** | **Related gene** | **Region** |  |
|  |  |  |  |  |  |  |  |  |  |  |
| 51 | 6 | rs9791189 | 11431851 | 5/22/36 | 19/40/26 | 11.79 | 5.96E-04 | *NEDD9* | 5' flanking |  |
| 52 | 12 | rs1996080 | 69657714 | 8/27/28 | 0/29/56 | 11.78 | 5.99E-04 | *PTPRR* | 5' flanking |  |
| 53 | 2 | rs12618935 | 13601818 | 2/26/35 | 1/14/70 | 11.78 | 6.00E-04 | *TRIB2* | 3' flanking |  |
| 54 | 8 | rs6997214 | 40019346 | 8/30/25 | 2/28/55 | 11.77 | 6.00E-04 | *INDOL1* | 3' flanking |  |
| 55 | 17 | rs2097727 | 28409856 | 0/12/51 | 0/2/83 | 11.77 | 6.01E-04 | *ACCN1* | intron |  |
| 56 | 15 | rs7172656 | 71843278 | 5/30/28 | 18/50/17 | 11.75 | 6.09E-04 | *LOC388135* | 5' flanking |  |
| 57 | 2 | rs1631149 | 236300113 | 15/31/17 | 8/32/45 | 11.67 | 6.35E-04 | *CENTG2* | intron |  |
| 58 | 12 | rs17021633 | 92772669 | 16/33/14 | 5/46/34 | 11.62 | 6.52E-04 | *CRADD* | 3' flanking |  |
| 59 | 19 | rs4801951 | 58274248 | 0/11/52 | 5/31/49 | 11.6 | 6.60E-04 | *ZNF160* | intron |  |
| 60 | 2 | rs4671389 | 60458534 | 5/25/33 | 12/54/19 | 11.56 | 6.73E-04 | *BCL11A* | 3' flanking |  |
| 61 | 3 | rs1010553 | 52515813 | 7/27/29 | 19/50/16 | 11.55 | 6.79E-04 | *STAB1* | syn^‡^ |  |
| 62 | 4 | rs6534608 | 76250676 | 2/15/46 | 0/6/79 | 11.53 | 6.84E-04 | *DKFZP564O0823* | 3' flanking |  |
| 63 | 4 | rs17010372 | 126995571 | 0/3/60 | 0/22/63 | 11.5 | 6.97E-04 | *LOC645841* | 5' flanking |  |
| 64 | 15 | rs4779858 | 29394407 | 6/32/25 | 23/46/16 | 11.44 | 7.20E-04 | *LOC643950* | 3' flanking |  |
| 65 | 18 | rs6506006 | 2341159 | 7/26/30 | 2/21/62 | 11.42 | 7.26E-04 | *METTL4* | 3' flanking |  |
| 66 | 4 | rs12643804 | 47943015 | 5/26/32 | 24/35/25 | 11.39 | 7.39E-04 | *TEC* | 5' flanking |  |
| 67 | 2 | rs7586253 | 60489196 | 1/20/42 | 5/47/33 | 11.33 | 7.64E-04 | *BCL11A* | 3' flanking |  |
| 68 | 1 | rs11207037 | 57677552 | 21/28/14 | 10/40/35 | 11.3 | 7.75E-04 | *DAB1* | intron |  |
| 69 | 1 | rs10737373 | 34030058 | 1/24/38 | 7/49/29 | 11.22 | 8.08E-04 | *CSMD2* | intron |  |
| 70 | 20 | rs4810051 | 54896305 | 9/28/26 | 29/39/17 | 11.19 | 8.22E-04 | *TFAP2C* | 3' flanking |  |
| 71 | 7 | rs4721740 | 19021805 | 6/29/28 | 21/45/18 | 11.11 | 8.56E-04 | *HDAC9* | 3' flanking |  |
| 72 | 16 | rs8058542 | 7794985 | 2/23/38 | 0/14/71 | 11.08 | 8.71E-04 | *A2BP1* | 3' flanking |  |
| 73 | 13 | rs11620374 | 36137211 | 1/15/47 | 7/37/41 | 11.06 | 8.80E-04 | *LOC400120* | 5' flanking |  |
| 74 | 1 | rs1338300 | 230523808 | 10/32/21 | 5/29/51 | 11.06 | 8.81E-04 | *SIPA1L2* | 3' flanking |  |
| 75 | 21 | rs2254368 | 42735535 | 20/34/9 | 13/41/31 | 11 | 9.11E-04 | *UBASH3A* | intron |  |
| 76 | 3 | rs10510551 | 24902207 | 1/12/50 | 6/34/45 | 10.97 | 9.28E-04 | *LOC442077* | 5' flanking |  |
| 77 | 16 | rs9941229 | 14401076 | 0/2/61 | 0/19/66 | 10.93 | 9.46E-04 | *PARN* | 3' flanking |  |
| 78 | 9 | rs7025094 | 100707447 | 16/33/14 | 9/37/39 | 10.9 | 9.62E-04 | *COL15A1* | 5' flanking |  |
| 79 | 8 | rs10283134 | 126341828 | 2/18/43 | 11/38/36 | 10.89 | 9.68E-04 | *NSMCE2* | intron |  |
| 80 | 2 | rs11689516 | 133911000 | 5/32/25 | 21/47/17 | 10.87 | 9.79E-04 | *NULL* | intron |  |
| 81 | 1 | rs2658399 | 63926240 | 5/35/23 | 21/49/15 | 10.79 | 1.02E-03 | *PGM1* | 3' flanking |  |
| 82 | 1 | rs4659786 | 235614223 | 13/28/22 | 5/31/49 | 10.74 | 1.05E-03 | *RYR2* | intron |  |
| 83 | 20 | rs6025312 | 55031807 | 1/4/58 | 2/26/57 | 10.7 | 1.07E-03 | *LOC728902* | 3' flanking |  |
| 84 | 17 | rs1730488 | 5026581 | 8/32/23 | 24/48/13 | 10.65 | 1.10E-03 | *ZNF594* | syn^‡^ |  |
| 85 | 9 | rs11794903 | 131039589 | 1/20/42 | 0/10/75 | 10.6 | 1.13E-03 | *IER5L* | 5' flanking |  |
| 86 | 7 | rs1358434 | 110230407 | 0/8/54 | 2/29/54 | 10.55 | 1.16E-03 | *IMMP2L* | intron |  |
| 87 | 1 | rs6541330 | 228950986 | 2/24/37 | 9/48/28 | 10.49 | 1.20E-03 | *CAPN9* | intron |  |
| 88 | 7 | rs6946475 | 24082848 | 21/32/10 | 12/44/29 | 10.47 | 1.21E-03 | *STK31* | intergenic |  |
| 89 | 18 | rs2069124 | 55236524 | 2/23/38 | 1/12/72 | 10.46 | 1.22E-03 | *CCBE1* | 3' flanking |  |
| 90 | 8 | rs2068674 | 72767933 | 10/32/21 | 3/35/47 | 10.41 | 1.26E-03 | *LOC729649* | 3' flanking |  |
| 91 | 12 | rs7965899 | 69584942 | 12/29/22 | 4/33/48 | 10.41 | 1.26E-03 | *PTPRR* | intron |  |
| 92 | 20 | rs2426694 | 55466125 | 3/23/37 | 17/37/31 | 10.4 | 1.26E-03 | *TFAP2C | BMP7* | intergenic |  |
| 93 | 7 | rs1554465 | 45730624 | 2/18/43 | 1/7/77 | 10.39 | 1.27E-03 | *40434* | 5' UTR |  |
| 94 | 21 | rs8127336 | 27297806 | 3/17/43 | 0/10/75 | 10.36 | 1.29E-03 | *ADAMTS5* | 5' flanking |  |
| 95 | 6 | rs6929649 | 107087881 | 13/33/17 | 4/42/39 | 10.36 | 1.29E-03 | *AIM1* | intron |  |
| 96 | 13 | rs4942192 | 42667263 | 17/35/11 | 12/37/36 | 10.34 | 1.30E-03 | *ENOX1* | 3' flanking |  |
| 97 | 22 | rs132518 | 37623718 | 1/19/43 | 6/43/36 | 10.34 | 1.30E-03 | *CBX6* | 5' flanking |  |
| 98 | 11 | rs10831300 | 94404981 | 1/25/37 | 0/15/69 | 10.34 | 1.31E-03 | *LOC643118* | intron |  |
| 99 | 7 | rs955056 | 104861024 | 3/17/43 | 8/43/33 | 10.33 | 1.31E-03 | *PUS7* | 3' flanking |  |
| 100 | 4 | rs11100087 | 158185329 | 0/16/46 | 5/37/42 | 10.33 | 1.31E-03 | *GLRB* | 5' flanking |  |
|  |  |  |  |  |  |  |  |  |  |  |
|  |  |  |  |  |  |  |  |  |  |  |
| **CHR, chromosome number; Position, chromosomal position (bp); Related gene, the nearest gene from the SNP site;** | | | | | | | | | |  |
| **^‡^, coding region (synonymous polymorphism);** | | | | |  |  |  |  |  |  |
| **^§^, distribution of genotype (homozygote of minor allele / heterozygote / homozygote of major allele)** | | | | | | | | |  |  |
