# Supplementary material for: Associations between the orexin (hypocretin) receptor 2 gene polymorphism Val308Ile and nicotine dependence in genome-wide and subsequent association studies
Source: Mol Brain. 2015 Aug 20;8:50. doi: 10.1186/s13041-015-0142-x (PMC4546081; doi:10.1186/s13041-015-0142-x)
Supplement: Additional file 2: Table S2. — Top 51–100 candidate SNPs possibly associated with nicotine dependence (TDS score). (DOC 127 kb) [file 13041_2015_142_MOESM2_ESM.doc]

| **Table S2. Top 51-100 candidate SNPs possibly associated with nicotine dependence (TDS score).** | | | | | | | | | | |
| --- | --- | --- | --- | --- | --- | --- | --- | --- | --- | --- |
|  |  |  |  |  |  |  |  |  |  |  |
| **Rank** | **CHR** | **SNP** | **Position** | **Genotype^§^ (TDS ≥ 3)** | **Genotype^§^ (TDS < 3)** | ***χ^2^*** | ***p*** | **Related gene** | **Region** |  |
|  |  |  |  |  |  |  |  |  |  |  |
| 51 | 11 | rs4758155 | 7043471 | 2/21/18 | 0/7/20 | 12.56 | 3.94E-04 | *NLRP14* | intron |  |
| 52 | 1 | rs11206608 | 39916684 | 3/21/17 | 9/12/6 | 12.56 | 3.95E-04 | *HPCAL4* | 3' flanking |  |
| 53 | 11 | rs10835823 | 31841755 | 5/25/11 | 2/8/17 | 12.55 | 3.95E-04 | *LOC729605* | 5' flanking |  |
| 54 | 11 | rs347767 | 96762331 | 3/19/18 | 0/7/20 | 12.52 | 4.02E-04 | *JRKL* | 3' flanking |  |
| 55 | 15 | rs11071240 | 54114319 | 14/18/9 | 3/10/14 | 12.38 | 4.34E-04 | *LOC729530* | 5' flanking |  |
| 56 | 14 | rs179539 | 30303410 | 0/12/29 | 0/1/26 | 12.37 | 4.37E-04 | *SCFD1* | 3' flanking |  |
| 57 | 9 | rs1953018 | 78937137 | 6/22/13 | 11/10/6 | 12.35 | 4.41E-04 | *VPS13A* | 5' flanking |  |
| 58 | 7 | rs10250905 | 37873829 | 6/15/20 | 8/15/4 | 12.35 | 4.42E-04 | *TXNDC3* | non-syn^†^ |  |
| 59 | 2 | rs7584554 | 233973521 | 8/20/13 | 11/13/3 | 12.29 | 4.55E-04 | *DGKD* | intron |  |
| 60 | 16 | rs8053948 | 81841906 | 5/19/17 | 1/7/19 | 12.29 | 4.55E-04 | *CDH13* | intron |  |
| 61 | 4 | rs3775793 | 37517001 | 0/11/30 | 4/11/12 | 12.28 | 4.58E-04 | *PGM2* | intron |  |
| 62 | 14 | rs8009561 | 37685769 | 5/17/19 | 0/9/18 | 12.26 | 4.64E-04 | *LOC729122* | intron |  |
| 63 | 5 | rs2561115 | 38384330 | 4/14/23 | 1/4/22 | 12.22 | 4.72E-04 | *EGFLAM* | intron |  |
| 64 | 13 | rs9542540 | 70411490 | 1/9/31 | 0/1/26 | 12.19 | 4.81E-04 | *LOC647277* | 3' flanking |  |
| 65 | 7 | rs17158049 | 110288215 | 0/11/30 | 0/1/26 | 12.19 | 4.81E-04 | *IMMP2L* | intron |  |
| 66 | 5 | rs10512781 | 41270400 | 2/26/13 | 0/12/15 | 12.16 | 4.89E-04 | *C6* | 5' flanking |  |
| 67 | 4 | rs4351004 | 24285706 | 2/10/29 | 0/1/26 | 12.15 | 4.91E-04 | *DHX15* | 5' flanking |  |
| 68 | 5 | rs12652381 | 169956120 | 7/22/12 | 3/5/19 | 12.09 | 5.06E-04 | *KCNIP1* | intron |  |
| 69 | 4 | rs4696669 | 8113102 | 0/1/40 | 0/6/21 | 12.05 | 5.19E-04 | *ABLIM2* | intron |  |
| 70 | 10 | rs7068239 | 83776831 | 0/1/40 | 1/5/21 | 12.04 | 5.20E-04 | *NRG3* | intron |  |
| 71 | 10 | rs2750050 | 33469928 | 0/4/37 | 1/7/19 | 12.04 | 5.20E-04 | *NRP1* | 3' flanking |  |
| 72 | 1 | rs6703086 | 237410351 | 4/20/17 | 0/8/19 | 12.01 | 5.30E-04 | *LOC729567* | 5' flanking |  |
| 73 | 15 | rs12594168 | 52210268 | 2/18/21 | 9/7/11 | 11.98 | 5.39E-04 | *UNC13C* | intron |  |
| 74 | 2 | rs6437268 | 240649535 | 5/17/19 | 7/14/6 | 11.92 | 5.55E-04 | *OR6B3* | 5' flanking |  |
| 75 | 12 | rs4134302 | 46197456 | 0/19/22 | 0/3/24 | 11.88 | 5.67E-04 | *LOC728148* | 3' flanking |  |
| 76 | 8 | rs1471610 | 3453250 | 5/20/16 | 10/12/5 | 11.86 | 5.73E-04 | *CSMD1* | intron |  |
| 77 | 4 | rs223374 | 103977443 | 6/25/10 | 1/13/13 | 11.83 | 5.83E-04 | *UBE2D3* | intron |  |
| 78 | 11 | rs7131435 | 7154630 | 5/20/16 | 0/9/18 | 11.78 | 5.97E-04 | *SYT9* | 5' flanking |  |
| 79 | 5 | rs13188039 | 84542188 | 10/23/8 | 4/11/12 | 11.77 | 6.02E-04 | *LOC402219* | 5' flanking |  |
| 80 | 6 | rs6907555 | 4484479 | 6/14/20 | 7/12/6 | 11.73 | 6.16E-04 | *KU-MEL-3* | 5' flanking |  |
| 81 | 3 | rs537932 | 175895898 | 4/24/13 | 8/15/4 | 11.72 | 6.17E-04 | *LOC647212* | 3' flanking |  |
| 82 | 15 | rs4774684 | 52212698 | 1/9/31 | 6/8/13 | 11.7 | 6.25E-04 | *UNC13C* | intron |  |
| 83 | 2 | rs6753628 | 47070598 | 0/12/29 | 0/16/11 | 11.68 | 6.31E-04 | *TTC7A* | intron |  |
| 84 | 16 | rs6499548 | 51899249 | 0/7/34 | 1/13/13 | 11.67 | 6.35E-04 | *CHD9* | non-syn^†^ |  |
| 85 | 2 | rs2271834 | 68862808 | 0/7/34 | 0/12/15 | 11.67 | 6.35E-04 | *ARHGAP25* | intron |  |
| 86 | 7 | rs4716763 | 155079733 | 0/10/31 | 4/10/13 | 11.64 | 6.45E-04 | *CNPY1* | 5' flanking |  |
| 87 | 3 | rs9311785 | 6109126 | 0/16/25 | 8/7/12 | 11.59 | 6.62E-04 | *GRM7* | 5' flanking |  |
| 88 | 22 | rs2247115 | 20354150 | 2/13/26 | 5/12/10 | 11.59 | 6.62E-04 | *PPIL2* | intron |  |
| 89 | 1 | rs12095036 | 198690464 | 4/17/20 | 9/10/8 | 11.57 | 6.69E-04 | *ZNF281* | 5' flanking |  |
| 90 | 14 | rs10459428 | 21363919 | 0/16/25 | 0/4/23 | 11.56 | 6.75E-04 | *TRAV10* | coding |  |
| 91 | 7 | rs17151577 | 78309085 | 2/15/24 | 5/12/10 | 11.53 | 6.84E-04 | *MAGI2* | intron |  |
| 92 | 10 | rs7092447 | 37159193 | 0/10/31 | 0/1/26 | 11.41 | 7.30E-04 | *LOC389948* | 3' flanking |  |
| 93 | 8 | rs3816747 | 13401173 | 7/23/11 | 0/13/14 | 11.41 | 7.31E-04 | *DLC1* | non-syn^†^ |  |
| 94 | 16 | rs2113294 | 81851629 | 6/15/20 | 0/8/19 | 11.35 | 7.54E-04 | *CDH13* | intron |  |
| 95 | 13 | rs9603226 | 37041586 | 5/21/15 | 0/12/15 | 11.34 | 7.57E-04 | *POSTN* | intron |  |
| 96 | 6 | rs9376770 | 143976398 | 5/22/14 | 9/13/5 | 11.33 | 7.62E-04 | *PHACTR2* | intron |  |
| 97 | 7 | rs6966980 | 147999887 | 2/13/26 | 6/11/10 | 11.33 | 7.63E-04 | *CUL1* | 5' flanking |  |
| 98 | 7 | rs6952125 | 6336719 | 1/8/32 | 0/1/26 | 11.31 | 7.70E-04 | *MGC12966* | non-syn^†^ |  |
| 99 | 18 | rs11152409 | 61604279 | 0/19/22 | 0/6/21 | 11.26 | 7.91E-04 | *SERPINB10 | HMSD* | intergenic |  |
| 100 | 2 | rs11126981 | 85249918 | 0/10/31 | 0/15/12 | 11.15 | 8.38E-04 | *TCF7L1* | intron |  |
|  |  |  |  |  |  |  |  |  |  |  |
|  |  |  |  |  |  |  |  |  |  |  |
| **CHR, chromosome number; Position, chromosomal position (bp); Related gene, the nearest gene from the SNP site;** | | | | | | | | | |  |
| **^†^, coding region (nonsynonymous polymorphism);** | | | | |  |  |  |  |  |  |
| **^§^, distribution of genotype (homozygote of minor allele / heterozygote / homozygote of major allele)** | | | | | | | | |  |  |
