# Supplementary material for: Associations between the orexin (hypocretin) receptor 2 gene polymorphism Val308Ile and nicotine dependence in genome-wide and subsequent association studies
Source: Mol Brain. 2015 Aug 20;8:50. doi: 10.1186/s13041-015-0142-x (PMC4546081; doi:10.1186/s13041-015-0142-x)
Supplement: Additional file 3: Table S3. — Top 51–100 candidate SNPs possibly associated with nicotine dependence (CPD). (DOC 126 kb) [file 13041_2015_142_MOESM3_ESM.doc]

| **Table S3. Top 51-100 candidate SNPs possibly associated with nicotine dependence (CPD).** | | | | | | | | | |  |
| --- | --- | --- | --- | --- | --- | --- | --- | --- | --- | --- |
|  |  |  |  |  |  |  |  |  |  |  |
| **Rank** | **CHR** | **SNP** | **Position** | **Genotype^§^ (CPD > 20)** | **Genotype^§^ (CPD ≤ 20)** | ***χ^2^*** | ***p*** | **Related gene** | **Region** |  |
|  |  |  |  |  |  |  |  |  |  |  |
| 51 | 8 | rs2470718 | 95428253 | 2/8/3 | 2/15/38 | 12.68 | 3.69E-04 | *LOC643022* | 5' flanking |  |
| 52 | 7 | rs6465948 | 103403034 | 4/8/1 | 8/21/26 | 12.63 | 3.80E-04 | *RELN* | intron |  |
| 53 | 8 | rs2166708 | 3264025 | 1/4/8 | 0/7/48 | 12.58 | 3.89E-04 | *CSMD1* | intron |  |
| 54 | 7 | rs13221086 | 88289441 | 1/4/8 | 8/32/15 | 12.57 | 3.92E-04 | *MGC26647* | 5' flanking |  |
| 55 | 4 | rs17040963 | 111023256 | 1/8/4 | 0/17/38 | 12.5 | 4.07E-04 | *KRT19P3* | 5' flanking |  |
| 56 | 13 | rs2992100 | 87077050 | 2/4/7 | 0/10/45 | 12.47 | 4.13E-04 | *SLITRK5* | 5' flanking |  |
| 57 | 13 | rs1867248 | 18432157 | 2/9/2 | 2/21/31 | 12.32 | 4.49E-04 | *LOC645666* | intron |  |
| 58 | 21 | rs1373484 | 21594668 | 0/5/8 | 8/32/15 | 12.29 | 4.55E-04 | *NCAM2* | intron |  |
| 59 | 7 | rs13437864 | 150153498 | 1/5/7 | 0/9/46 | 12.17 | 4.87E-04 | *TMEM176A* | 3' flanking |  |
| 60 | 12 | rs2727733 | 118218257 | 3/9/1 | 5/18/32 | 12.13 | 4.95E-04 | *CCDC60* | 5' flanking |  |
| 61 | 2 | rs9678322 | 33417079 | 2/11/0 | 6/23/26 | 12.08 | 5.08E-04 | *LTBP1* | intron |  |
| 62 | 7 | rs7795788 | 122932940 | 1/6/6 | 20/28/7 | 12.07 | 5.13E-04 | *SLC13A1 | IQUB* | intergenic |  |
| 63 | 11 | rs10837645 | 1592388 | 1/5/7 | 0/11/44 | 12.03 | 5.23E-04 | *KRTAP5-3* | 5' flanking |  |
| 64 | 11 | rs7932326 | 43949600 | 1/3/9 | 1/3/51 | 12.02 | 5.25E-04 | *LOC387763* | 3' flanking |  |
| 65 | 13 | rs913798 | 28191681 | 0/6/7 | 0/7/48 | 11.91 | 5.57E-04 | *SLC46A3* | 5' flanking |  |
| 66 | 11 | rs188311 | 85084563 | 0/1/12 | 5/22/27 | 11.9 | 5.62E-04 | *SYTL2* | intron |  |
| 67 | 7 | rs10245818 | 129044744 | 6/6/1 | 12/28/15 | 11.86 | 5.72E-04 | *NRF1* | intron |  |
| 68 | 9 | rs7047899 | 21847303 | 1/7/5 | 1/13/41 | 11.84 | 5.80E-04 | *MTAP* | intron |  |
| 69 | 2 | rs6544318 | 40381169 | 2/8/3 | 3/22/30 | 11.82 | 5.87E-04 | *SLC8A1* | intron |  |
| 70 | 1 | rs703801 | 14536213 | 3/9/1 | 7/24/24 | 11.74 | 6.13E-04 | *RP1-21O18.1* | 5' flanking |  |
| 71 | 5 | rs12153251 | 29087637 | 1/5/7 | 0/9/46 | 11.65 | 6.42E-04 | *LOC729862* | 3' flanking |  |
| 72 | 11 | rs10892480 | 119242858 | 1/5/7 | 18/25/12 | 11.63 | 6.47E-04 | *LOC390255* | 5' flanking |  |
| 73 | 15 | rs2170878 | 78802036 | 0/6/7 | 1/7/47 | 11.62 | 6.54E-04 | *FAM108C1* | intron |  |
| 74 | 1 | rs7551386 | 64800419 | 1/5/7 | 0/9/45 | 11.61 | 6.55E-04 | *CACHD1* | intron |  |
| 75 | 2 | rs10176321 | 235561801 | 0/6/7 | 16/28/11 | 11.58 | 6.66E-04 | *SH3BP4* | intron |  |
| 76 | 12 | rs964447 | 24769858 | 0/3/10 | 0/2/53 | 11.54 | 6.81E-04 | *BCAT1* | 3' flanking |  |
| 77 | 21 | rs2836902 | 39421337 | 0/2/11 | 4/23/28 | 11.52 | 6.88E-04 | *LOC391282* | intron |  |
| 78 | 6 | rs9366472 | 22800984 | 0/4/9 | 0/4/51 | 11.51 | 6.93E-04 | *LOC389370* | intron |  |
| 79 | 5 | rs6894296 | 179465550 | 2/7/4 | 2/14/39 | 11.51 | 6.94E-04 | *RASGEF1C* | intron |  |
| 80 | 16 | rs444163 | 4160304 | 1/5/7 | 15/25/15 | 11.49 | 7.00E-04 | *SRL* | 3' flanking |  |
| 81 | 8 | rs987231 | 122349522 | 1/3/9 | 8/28/19 | 11.47 | 7.06E-04 | *HAS2* | 3' flanking |  |
| 82 | 18 | rs6567322 | 58886429 | 0/4/9 | 12/28/15 | 11.47 | 7.07E-04 | *BCL2* | 3' flanking |  |
| 83 | 13 | rs12872598 | 111618993 | 5/8/0 | 13/22/20 | 11.46 | 7.13E-04 | *LOC729095* | 3' flanking |  |
| 84 | 4 | rs16893797 | 16303961 | 1/6/6 | 2/11/42 | 11.42 | 7.27E-04 | *LDB2* | intron |  |
| 85 | 13 | rs7999688 | 100817298 | 1/7/5 | 1/12/42 | 11.38 | 7.43E-04 | *NALCN* | intron |  |
| 86 | 5 | rs2240784 | 149555895 | 0/5/8 | 12/23/20 | 11.37 | 7.45E-04 | *SLC6A7* | intron |  |
| 87 | 1 | rs2182626 | 182212070 | 0/3/10 | 10/26/19 | 11.34 | 7.57E-04 | *GLT25D2* | intron |  |
| 88 | 16 | rs13330807 | 19809639 | 5/8/0 | 11/18/26 | 11.34 | 7.61E-04 | *GPRC5B* | 5' flanking |  |
| 89 | 10 | rs1561439 | 80368206 | 1/7/3 | 1/20/33 | 11.31 | 7.72E-04 | *ZMIZ1* | 5' flanking |  |
| 90 | 8 | rs12235009 | 28547337 | 3/4/6 | 1/17/37 | 11.3 | 7.75E-04 | *LOC730056* | 3' flanking |  |
| 91 | 21 | rs171216 | 27682530 | 0/6/7 | 11/33/11 | 11.29 | 7.81E-04 | *ADAMTS5* | 5' flanking |  |
| 92 | 8 | rs2200015 | 79153384 | 6/5/2 | 8/29/18 | 11.28 | 7.86E-04 | *PKIA* | 5' flanking |  |
| 93 | 2 | rs17426403 | 98129591 | 2/6/5 | 0/18/37 | 11.26 | 7.92E-04 | *VWA3B* | intron |  |
| 94 | 7 | rs12113884 | 88376429 | 2/9/2 | 0/28/27 | 11.26 | 7.94E-04 | *MGC26647* | 5' flanking |  |
| 95 | 12 | rs10748046 | 65153840 | 0/7/6 | 0/9/46 | 11.24 | 8.00E-04 | *GRIP1* | intron |  |
| 96 | 20 | rs6062530 | 61888829 | 1/5/7 | 0/13/42 | 11.2 | 8.20E-04 | *ZBTB46* | intron |  |
| 97 | 8 | rs16908616 | 139300665 | 0/0/13 | 1/13/41 | 11.18 | 8.28E-04 | *FAM135B* | 5' flanking |  |
| 98 | 7 | rs2237419 | 42068497 | 0/0/13 | 2/19/34 | 11.14 | 8.43E-04 | *GLI3* | intron |  |
| 99 | 9 | rs10758211 | 33508281 | 1/3/9 | 0/5/50 | 11.13 | 8.51E-04 | *ANKRD18B* | 5' flanking |  |
| 100 | 10 | rs7071875 | 52512361 | 0/1/12 | 1/20/34 | 11.09 | 8.66E-04 | *PRKG1* | intron |  |
|  |  |  |  |  |  |  |  |  |  |  |
|  |  |  |  |  |  |  |  |  |  |  |
| **CHR, chromosome number; Position, chromosomal position (bp); Related gene, the nearest gene from the SNP site;** | | | | | | | | | |  |
| **^§^, distribution of genotype (homozygote of minor allele / heterozygote / homozygote of major allele)** | | | | | | | | |  |  |
