# Supplementary material for: Associations between the orexin (hypocretin) receptor 2 gene polymorphism Val308Ile and nicotine dependence in genome-wide and subsequent association studies
Source: Mol Brain. 2015 Aug 20;8:50. doi: 10.1186/s13041-015-0142-x (PMC4546081; doi:10.1186/s13041-015-0142-x)
Supplement: Additional file 4: Table S4. — SNPs or genes commonly included in the GWAS candidates for different phenotypes. (DOC 174 kb) [file 13041_2015_142_MOESM4_ESM.doc]

| **Table S4. SNPs or genes commonly included in the GWAS candidates for different phenotypes.** | | | | | | | | | | |  |
| --- | --- | --- | --- | --- | --- | --- | --- | --- | --- | --- | --- |
|  |  |  |  |  |  |  |  |  |  |  |  |
| ***1** | ***2** | ***3** | ***4** | **Phenotype** | **Rank** | **CHR** | **SNP** | ***p*** | **Related gene** | **Region** | **Reference** |
|  |  |  |  |  |  |  |  |  |  |  |  |
| + | + |  |  | FTND | 209 | 8 | rs1523254 | 0.004787 | *CSMD1* | intron | (10-14) |
| + | + |  |  | TDS | 76 | 8 | rs1471610 | 0.0005726 | *CSMD1* | intron | (10-14) |
| + | + |  |  | CPD | 53 | 8 | rs2166708 | 0.0003889 | *CSMD1* | intron | (10-14) |
| + | + |  |  | FTND | 86 | 7 | rs1358434 | 0.001163 | *IMMP2L* | intron |  |
| + | + |  |  | TDS | 65 | 7 | rs17158049 | 0.0004807 | *IMMP2L* | intron |  |
| + | + |  |  | CPD | 194 | 7 | rs4730439 | 0.003141 | *IMMP2L* | 3' flanking |  |
|  | + |  |  | FTND | 72 | 16 | rs8058542 | 0.0008714 | *A2BP1* | 3' flanking | (10-13) |
| + |  |  |  | CPD | 164 | 16 | rs12923619 | 0.002317 | *A2BP1* | intron | (10-13) |
| + |  |  |  | CPD | 222 | 16 | rs1994766 | 0.004711 | *A2BP1* | intron | (10-13) |
|  | + |  | + | FTND | 94 | 21 | rs8127336 | 0.001287 | *ADAMTS5* | 5' flanking |  |
| + |  |  |  | CPD | 20 | 21 | rs2830596 | 0.0001623 | *ADAMTS5* | 5' flanking |  |
| + |  | + |  | CPD | 9 | 21 | rs8127336 | 0.00003591 | *ADAMTS5* | 5' flanking |  |
| + |  |  |  | CPD | 91 | 21 | rs171216 | 0.0007807 | *ADAMTS5* | 5' flanking |  |
| + |  |  |  | FTND | 38 | 2 | rs17028222 | 0.0004033 | *BCL11A* | 3' flanking |  |
| + |  |  |  | FTND | 60 | 2 | rs4671389 | 0.000673 | *BCL11A* | 3' flanking |  |
| + |  |  |  | FTND | 67 | 2 | rs7586253 | 0.000764 | *BCL11A* | 3' flanking |  |
| + |  |  |  | TDS | 209 | 2 | rs1468739 | 0.003711 | *BCL11A* | 3' flanking |  |
|  | + |  |  | TDS | 163 | 9 | rs9406647 | 0.002089 | *BNC2* | 3' flanking | (11) |
|  | + |  |  | CPD | 191 | 9 | rs6475020 | 0.00296 | *BNC2* | 3' flanking | (11) |
|  | + |  |  | FTND | 146 | 18 | rs12458388 | 0.001991 | *C18orf20* | 5' flanking |  |
| + |  |  |  | CPD | 182 | 18 | rs2193860 | 0.002767 | *C18orf20* | 5' flanking |  |
| + |  |  |  | CPD | 248 | 18 | rs1592717 | 0.01084 | *C18orf20* | 5' flanking |  |
|  | + |  |  | FTND | 257 | 9 | rs7858008 | 0.01054 | *C9orf27* | 3' flanking |  |
| + |  |  |  | CPD | 193 | 9 | rs966955 | 0.003125 | *C9orf27* | 3' flanking |  |
| + |  |  |  | FTND | 111 | 5 | rs1169653 | 0.001498 | *CDH10* | 5' flanking |  |
| + |  |  |  | TDS | 122 | 5 | rs1169654 | 0.001105 | *CDH10* | 5' flanking |  |
|  | + |  |  | TDS | 132 | 12 | rs12579246 | 0.001238 | *FAM19A2* | 3' flanking |  |
|  | + |  |  | CPD | 138 | 12 | rs7973414 | 0.001671 | *FAM19A2* | 3' flanking |  |
| + |  |  |  | FTND | 215 | 11 | rs831596 | 0.005063 | *FBXO3* | intron |  |
| + |  |  |  | TDS | 120 | 11 | rs953871 | 0.001076 | *FBXO3* | intron |  |
|  | + |  | + | FTND | 105 | 15 | rs10152981 | 0.001366 | *GABRG3* | 5' flanking |  |
| + |  | + |  | CPD | 246 | 15 | rs10152981 | 0.009605 | *GABRG3* | 5' flanking |  |
|  | + |  |  | FTND | 49 | 7 | rs10230426 | 0.0005657 | *HDAC9* | intron |  |
|  | + |  |  | FTND | 71 | 7 | rs4721740 | 0.0008564 | *HDAC9* | 3' flanking |  |
| + |  |  |  | CPD | 25 | 7 | rs3814991 | 0.0001802 | *HDAC9* | intron |  |
| + |  | + |  | FTND | 179 | 10 | rs520887 | 0.003409 | *KCNMA1* | intron |  |
| + |  |  |  | TDS | 153 | 10 | rs11002139 | 0.001902 | *KCNMA1* | intron |  |
| + |  |  |  | TDS | 41 | 10 | rs520887 | 0.0003146 | *KCNMA1* | intron |  |
|  | + |  |  | TDS | 80 | 6 | rs6907555 | 0.0006157 | *KU-MEL-3* | 5' flanking |  |
|  | + |  |  | CPD | 147 | 6 | rs234427 | 0.001854 | *KU-MEL-3* | 3' flanking |  |
|  | + |  | + | FTND | 116 | 1 | rs1416127 | 0.001594 | *LOC127406* | 3' flanking |  |
| + |  | + |  | CPD | 23 | 1 | rs1416127 | 0.0001718 | *LOC127406* | 3' flanking |  |
| + |  |  |  | FTND | 159 | 3 | rs2687863 | 0.002688 | *LOC389156* | 3' flanking |  |
| + |  |  |  | FTND | 35 | 3 | rs6807108 | 0.0003599 | *LOC389156* | 3' flanking |  |
| + |  |  |  | TDS | 137 | 3 | rs10513261 | 0.001418 | *LOC389156* | 3' flanking |  |
|  | + |  | + | FTND | 148 | 11 | rs10892480 | 0.002054 | *LOC390255* | 5' flanking |  |
| + |  | + |  | CPD | 72 | 11 | rs10892480 | 0.0006473 | *LOC390255* | 5' flanking |  |
|  | + |  |  | FTND | 40 | 21 | rs2242936 | 0.0004198 | *LOC391282* | 5' flanking |  |
| + |  |  |  | CPD | 101 | 21 | rs2836916 | 0.0008783 | *LOC391282* | 3' flanking |  |
| + |  |  |  | CPD | 77 | 21 | rs2836902 | 0.0006879 | *LOC391282* | intron |  |
| + |  |  |  | FTND | 212 | 5 | rs12520206 | 0.004839 | *LOC391849* | 5' flanking |  |
| + |  |  |  | TDS | 164 | 5 | rs6894799 | 0.002105 | *LOC391849* | 5' flanking |  |
|  | + |  |  | TDS | 219 | 1 | rs881713 | 0.004139 | *LOC729977* | 5' flanking |  |
|  | + |  |  | CPD | 160 | 1 | rs2813886 | 0.002246 | *LOC729977* | 5' flanking |  |
|  | + |  | + | FTND | 15 | 2 | rs1542604 | 0.0001422 | *LOC730032* | 5' flanking |  |
| + |  | + |  | CPD | 12 | 2 | rs1542604 | 0.00005261 | *LOC730032* | 5' flanking |  |
|  | + |  | + | FTND | 147 | 2 | rs9678322 | 0.002007 | *LTBP1* | intron |  |
| + |  | + |  | CPD | 61 | 2 | rs9678322 | 0.0005083 | *LTBP1* | intron |  |
|  |  | + |  | TDS | 179 | 7 | rs371004 | 0.002665 | *MDFIC* | 3' flanking |  |
| + |  |  |  | FTND | 115 | 10 | rs7902158 | 0.001569 | *NRG3* | intron | (11) |
| + |  |  |  | FTND | 168 | 10 | rs4933814 | 0.003004 | *NRG3* | intron | (11) |
| + |  |  |  | FTND | 219 | 10 | rs4933811 | 0.005462 | *NRG3* | intron | (11) |
| + |  |  |  | TDS | 70 | 10 | rs7068239 | 0.0005198 | *NRG3* | intron | (11) |
|  | + |  | + | FTND | 10 | 2 | rs10176321 | 0.0001075 | *SH3BP4* | intron |  |
|  | + |  |  | FTND | 14 | 2 | rs2042831 | 0.0001382 | *SH3BP4* | 5' flanking |  |
| + |  | + |  | CPD | 75 | 2 | rs10176321 | 0.0006655 | *SH3BP4* | intron |  |
| + |  |  |  | CPD | 48 | 25 | rs5988531 | 0.0003482 | *SHOX | CRLF2* | intergenic |  |
|  | + |  |  | FTND | 235 | 25 | rs28647890 | 0.007054 | *SHOX | CRLF2* | intergenic |  |
|  | + |  |  | TDS | 73 | 15 | rs12594168 | 0.0005388 | *UNC13C* | intron | (11) |
|  | + |  |  | TDS | 82 | 15 | rs4774684 | 0.0006251 | *UNC13C* | intron | (11) |
|  | + |  |  | CPD | 7 | 15 | rs10518761 | 0.0000197 | *UNC13C* | intron | (11) |
|  | + |  |  | FTND | 248 | 4 | rs992852 | 0.008579 | *UNC5C* | 5' flanking |  |
| + |  |  |  | CPD | 173 | 4 | rs13112339 | 0.002493 | *UNC5C* | 5' flanking |  |
| + |  |  |  | FTND | 222 | 7 | rs4730775 | 0.005909 | *WNT2* | 3' flanking |  |
| + |  |  |  | TDS | 178 | 7 | rs12669592 | 0.002632 | *WNT2* | 3' flanking |  |
|  | + |  | + | FTND | 236 | 10 | rs2275283 | 0.007104 | *ZMIZ1* | 5' flanking |  |
| + |  |  |  | CPD | 220 | 10 | rs9633597 | 0.004286 | *ZMIZ1* | 5' flanking |  |
| + |  | + |  | CPD | 227 | 10 | rs2275283 | 0.005346 | *ZMIZ1* | 5' flanking |  |
| + |  |  |  | CPD | 89 | 10 | rs1561439 | 0.0007717 | *ZMIZ1* | 5' flanking |  |
|  |  |  |  |  |  |  |  |  |  |  |  |
|  |  |  |  |  |  |  |  |  |  |  |  |
| **CHR, chromosome number; Related gene, the nearest gene from the SNP site;** | | | | | | | |  |  |  |  |
| ***1, Candidate genes commonly included in the GWAS candidates for FTND (for TDS/CPD candidates) or TDS (for FTND/CPD candidates);** | | | | | | | | | |  |  |
| ***2, Candidate genes commonly included in the GWAS candidates for TDS (for FTND/CPD candidates) or CPD (for FTND/TDS candidates);** | | | | | | | | | |  |  |
| ***3, Candidate SNPs commonly included in the GWAS candidates for FTND (for TDS/CPD candidates) or TDS (for FTND/CPD candidates);** | | | | | | | | | |  |  |
| ***4, Candidate SNPs commonly included in the GWAS candidates for TDS (for FTND/CPD candidates) or CPD (for FTND/TDS candidates)** | | | | | | | | | |  |  |
