# Supplementary material for: Associations between the orexin (hypocretin) receptor 2 gene polymorphism Val308Ile and nicotine dependence in genome-wide and subsequent association studies
Source: Mol Brain. 2015 Aug 20;8:50. doi: 10.1186/s13041-015-0142-x (PMC4546081; doi:10.1186/s13041-015-0142-x)
Supplement: Additional file 5: — Supporting information (Materials and Methods). (DOC 124 kb) [file 13041_2015_142_MOESM5_ESM.doc]

**Supporting information (Materials and Methods)**

Associations between the orexin (hypocretin) receptor 2 gene polymorphism Val308Ile and nicotine dependence in genome-wide and subsequent association studies

**Daisuke Nishizawa, Shinya Kasai, Junko Hasegawa, Naomi Sato, Fumihiko Tanioka, Makoto Nagashima, Ryoji Katoh, Yasuo Satoh, Megumi Tagami, Hiroshi Ujike, Norio Ozaki, Toshiya Inada, Nakao Iwata, Ichiro Sora, Masaomi Iyo, Mitsuhiko Yamada, Naoki Kondo, Moo-Jun Won, Nobuya Naruse, Kumi Uehara-Aoyama, Masanari Itokawa, Kazutaka Ohi, Ryota Hashimoto, Kumpei Tanisawa, Tomio Arai, Seijiro Mori, Motoji Sawabe, Makiko Naka–Mieno, Yoshiji Yamada, Miki Yamada, Noriko Sato, Masaaki Muramatsu, Masashi Tanaka, Yoko Irukayama-Tomobe, Yuki C Saito, Takeshi Sakurai, Masakazu Hayashida, Haruhiko Sugimura, & Kazutaka Ikeda**

**Subjects, surgery, and clinical data**

*Patients with smoking behavior data*

Subjects

The two-stage genome-wide association study (GWAS) to explore the association between polymorphisms in the entire human genome and the severity of nicotine dependence was conducted in 148 subjects among a total of 999 patients who visited Iwata City Hospital in Japan and were recruited in the study. The inclusion criteria included being Japanese, ambulatory, able to communicate orally, and 60 years of age or older. Numerous participants in this study had various smoking habits and completed a questionnaire that consisted of various questions about lifestyle, including alcohol consumption, smoking, diet, and cancer history (1, 2). Peripheral blood samples were collected from these subjects for the gene analysis. The study protocol was approved by the Institutional Review Boards at the Iwata City Hospital, Iwata, Japan, Hamamatsu University School of Medicine, Hamamatsu, Japan, and Tokyo Institute of Psychiatry (currently Tokyo Metropolitan Institute of Medical Science), Tokyo, Japan. All of the subjects provided informed, written consent for the genetic studies. The detailed demographic and clinical characteristics of the subjects are provided in Additional file 12: Table S11.

Smoking behavior data

For the subjects included in the study on the severity of nicotine dependence, the results of the questionnaire, especially the questions related to smoking, were used in the analysis. Some of the questions were from the Fagerstrӧm Test for Nicotine Dependence (FTND; a test that yields a continuous measure of nicotine dependence) and Tobacco Dependence Screener (TDS; a screening questionnaire for tobacco/nicotine dependence according to the *International Statistical Classiﬁcation of Diseases and Related Health Problems*, 10th revision [ICD-10], *Diagnostic and Statistical Manual of Mental Disorders*, 3rd edition [DSM-III-R], and DSM-IV), which consists of 10 questions (4). The questionnaire also included questions about the numbers of cigarettes smoked per day (CPD), the participants’ age when they began smoking, how many times current-smokers tried to quit smoking (i.e., the number of trials for smoking cessation in current-smokers), and how many times ex-smokers tried to quit smoking before succeeding (i.e., the number of trials for smoking cessation in ex-smokers). In the present study, the FTND, TDS, and CPD were used as measures of nicotine dependence (Additional file 12: Table S11).

*Patients who underwent major abdominal surgery*

Subjects

The recruitment of the subjects and basic protocol for postoperative pain management were described in a previous report (5, 6). The subjects used in the current association study included 112 Japanese patients who underwent major open abdominal surgery (age, 28-80 years; 60 males and 52 females), mostly gastrectomy for gastric cancer and colectomy for colorectal cancer under combined general and epidural anesthesia at the Research Hospital, Institute of Medical Science, The University of Tokyo, or Toho University Sakura Medical Center. Peripheral blood or oral mucosa samples were collected from these subjects for gene analysis. Patients who underwent surgery without substantially severe pain, such as laparoscopy-assisted distal gastrectomy (LADG), were excluded from the analyses. The study protocol was approved by the Institutional Review Boards at the Institute of Medical Science, The University of Tokyo, Tokyo, Japan, Toho University Sakura Medical Center, Sakura, Japan, and Tokyo Institute of Psychiatry (currently Tokyo Metropolitan Institute of Medical Science), Tokyo, Japan. All of the subjects provided informed, written consent for the genetic studies. The detailed demographic and clinical data of the subjects are provided in Additional file 6: Table S5.

Clinical data

Postoperative pain was managed primarily with continuous epidural analgesia with fentanyl or morphine. Fentanyl or morphine was diluted with 0.25% bupivacaine in a total volume of 100 ml and infused at a constant rate of 2 ml/h through a catheter placed in the lower thoracic or upper lumbar epidural space. Whenever the patient complained of significant postoperative pain despite continuous epidural analgesic administration, appropriate doses of opioids, including morphine, buprenorphine, pentazocine, and pethidine, or nonsteroidal antiinflammatory drugs (NSAIDs), including diclofenac and flurbiprofen, were administered as rescue analgesics at the discretion of the surgeons. The clinical data that were collected in the present study included age, gender, height, body weight, postoperative diagnosis, type of operation, duration of operation, and doses of rescue analgesics (opioids or NSAIDs) administered during the first 24-h postoperative period, for which analgesic therapy would be required in most patients. The study subjects were also asked to rate their pain intensity at rest during the first 24-h postoperative period using a 5-point verbal numerical rating scale (NRS; 0 = no pain, 1 = mild pain, 2 = moderate pain, 3 = severe pain, 4 = extremely severe pain).

To allow intersubject comparisons of rescue analgesic doses required during the first 24-h postoperative period, the doses of opioids and NSAIDs administered as rescue analgesics during this period were converted to the equivalent dose of systemic fentanyl according to previous reports (5, 6). The frequency of rescue analgesic administration was determined as the frequency of use of rescue analgesics during the first 24-h postoperative period. The total dose of rescue analgesics administered was calculated as the sum of systemic fentanyl-equivalent doses of all opioids and NSAIDs administered to patients as rescue analgesics during the same period. Doses of rescue analgesics administered postoperatively were normalized to body weight.

*Patients with methamphetamine dependence/psychosis*

Subjects

Enrolled in the study were 203 Japanese patients with methamphetamine (METH) dependence (age, 18-69 years; 165 males and 38 females), mostly with comorbid METH-induced psychosis (age, 19-69 years; 155 males and 30 females). Patients with an individual or family history of drug dependence or major psychotic disorders, such as schizophrenia and bipolar disorder, were excluded. Consensus of the diagnosis of METH dependence was made by at least two trained psychiatrists according to the criteria of the *International Classification of Diseases* (ICD) or *Diagnostic and Statistical Manual of Mental Disorders*, 4th edition (DSM-IV), based on interviews and medical records. Abusers who used METH but displayed no dependence/psychosis were excluded from the analyses. All of the subjects were unrelated Japanese who lived in Japan. The study was approved by the Institutional Review Board at the Tokyo Institute of Psychiatry (currently Tokyo Metropolitan Institute of Medical Science) and the Ethics Committee of each participating institute of the Japanese Genetics Initiative for Drug Abuse (JGIDA) (7, 8). Written informed consent was obtained from all of the patients and control subjects. The detailed demographic and clinical data of the patient subjects are provided in Additional file 7: Table S6.

Clinical data

A large number of patients were dependent not only on METH but also other drugs, such as cannabinoids, cocaine, lysergic acid diethylamide, and opioids. Given that patients with METH dependence/psychosis were divided into subgroups according to their multisubstance abuse status to estimate the severity of and liability to dependence, a total of 142 patients were polydrug abusers, and 54 patients were exclusively dependent on METH. Additionally, age at first use of the drug (years) was also recorded for each patient (Additional file 7: Table S6). The characteristics of the subjects used in this study have also been described in several other reports (9-11).

*Healthy subjects with* *schizotypal personality trait data*

Subjects

Data for the schizotypal personality trait analysis were available for 311 healthy subjects (41.5% male [129/182]; mean age ± SD: 36.4 ± 12.4 years). The subjects were all biologically unrelated and Japanese. The subjects were recruited through local advertisements at Osaka University. Psychiatrically, medically, and neurologically healthy subjects were evaluated using the Structured Clinical Interview for DSM-IV-Non-Patient Edition (SCID-I/NP) to exclude individuals who had received psychiatric medications or who had first- or second-degree relatives with psychiatric disorders. Additionally, subjects were excluded from this study if they had neurological or medical conditions that could potentially affect their central nervous system, such as atypical headaches, head trauma with loss of consciousness, chronic lung disease, kidney disease, chronic hepatic disease, thyroid disease, active cancer, cerebrovascular disease, epilepsy, seizures, substance-related disorders, or mental retardation. Demographic variables, age, sex, years of education, and the full-scale Intelligence Quotient (IQ) are provided in Additional file 8: Table S7. Age and years of education were significantly different between genotype groups (*p* < 0.05). Written informed consent was obtained from all of the subjects after the procedures were fully explained to them. This study was performed in accordance with the World Medical Association’s Declaration of Helsinki and approved by the Osaka University Research Ethics Committee.

Schizotypal personality trait data

To assess schizotypal personality traits, a full Japanese version of the Schizotypal Personality Questionnaire (SPQ) was administered to healthy subjects (12, 13). The SPQ is a 74-item self-report questionnaire with a “yes/no” response format (14). All of the “yes” answers received a score of 1. The SPQ measures nine subscales of specific schizotypal features. The total SPQ score was obtained by summing the scores from all of the items. The three schizotypal trait factors—cognitive/perceptual, interpersonal, and disorganization—were derived by summing the related subscale raw scores according to the three-factor model of Raine and colleagues (15). The full-scale IQ was assessed using the Wechsler Adult Intelligence Scale, Revised or Third edition (16).

*Samples from autopsy specimens*

Subjects

The study group initially comprised 2,305 consecutive Japanese autopsy cases at Tokyo Metropolitan Geriatric Hospital between 1995 and 2012 (1,266 men and 1,039 women; mean age at death: 80.6 ± 8.8 years). The subjects were registered in the Internet database of Japanese SNPs for geriatric research (17). Autopsy procedures were performed on approximately 40% of patients who died at the hospital. The presence or absence of any diseases was determined by thorough examination of the autopsy reports. Part of the pathological findings can be found in the Japanese SNPs for geriatric research database. All of the subjects provided informed, written consent for the genetic studies. The detailed demographic data of the patient subjects are provided in Additional file 10: Table S9.

Clinical data

The disease status of the study group was recorded based primarily on the pathological findings of the autopsies. The status was also recorded based on clinical diagnosis, and patient records were used for the analyses in the present study for subjects whose pathological findings were unavailable. As a result, the presence or absence of a total of 100 diseases were recorded and incorporated in the association analyses. Ancillary data were also collected for most of the subjects, such as alcohol drinking and smoking habits. The detailed clinical data of the patient subjects, including the ancillary data, are provided in Additional file 10: Table S9. Among 2,305 subjects, seven pairs of 14 samples were suspected to be derived from identical individuals and were removed from the demographic statistics and association analysis.

**Genotyping**

*whole-genome genotyping*

Genotyping procedure

A total of 300 DNA samples from the patients with smoking behavior data who visited Iwata City Hospital were used for genotyping. Total genomic DNA was extracted from whole-blood samples using a QIAamp DNA BloodMaxi kit according to the manufacturer’s instructions (Qiagen, Hamburg, Germany) and extracted from oral mucosa samples as described in a previous report (18). The extracted DNA was dissolved in TE buffer (10 mM Tris-HCl and 1 mM ethylenediaminetetraacetic acid [EDTA], pH 8.0). The DNA concentration was adjusted to 100 ng/μl for whole-genome genotyping using a NanoDrop ND-1000 Spectrophotometer (NanoDrop Technologies, Wilmington, DE, USA).

Briefly, whole-genome genotyping was performed using the Infinium assay II with an iScan system (Illumina, San Diego, CA, USA) according to the manufacturer’s instructions. HumanCytoSNP-12 v2.0 (total markers: 301,232) BeadChips were used for genotyping the 300 samples. The BeadChips included a number of probes that were specific to copy number variation markers, but most of the BeadChips were for SNP markers on the human autosome or sex chromosome.

Quality control

The data for the whole-genome genotyping samples were analyzed using GenomeStudio software with the Genotyping module v3.3.7 (Illumina) to evaluate the quality of the results. In the data-cleaning process, the samples with a genotype call rate of less than 0.95 were expected to be excluded from further analysis. As a result, no sample was excluded from further analysis. Markers with a genotype call frequency of less than 0.95 or “Cluster sep” (i.e., an index of genotype cluster separation) of less than 0.1 were excluded from the subsequent association study. A total of 225,602 SNP markers survived the filtration process and were used for the GWAS. Markers were excluded based on the results of Fisher’s exact tests that compared the distributions of the obtained genotype data with the theoretical distributions expected from Hardy-Weinberg equilibrium (HWE) in the initial step, confirming that significant deviation of the observed distributions from the expected distributions was observed for 11 SNPs, in which markers with *p* values (df = 1) greater than approximately 2 × 10^-7^ (0.05 / 225,602) were considered significantly deviated. Tests for autosomal heterozygosity and relatedness (identical-by-state [IBS]) were not conducted because all of the subjects were assumed to be unrelated and genetically homogeneous Japanese, mostly living in the Kanto or Tokai area. Therefore, a total of 225,591 SNP markers were used for the GWAS (Additional file 13: Figure S1).

*Genotyping for specific SNPs*

Genotyping procedure for specific SNPs

To genotype the rs2653349 and rs726016 SNPs in the subsequent association study after the GWAS, the TaqMan allelic discrimination assay (Life Technologies, Carlsbad, CA, USA) was basically adopted. A total of up to 699, 112, and 203 DNA samples from patients with smoking behavior data, patients who underwent major abdominal surgery, and patients with METH dependence/psychosis, respectively, were used for genotyping the rs2653349 SNP. The genotype distribution of the rs2653349 SNP in these subjects is provided in Additional file 14: Table S12.

For all of these samples, total genomic DNA was extracted from whole-blood or oral mucosa samples using standard procedures or as described in a previous report (18). The extracted DNA was dissolved in TE buffer (10 mM Tris-HCl and 1 mM EDTA, pH 8.0) before use. The DNA concentration was adjusted to approximately 5-50 ng/μl for genotyping the SNPs using a NanoDrop ND-1000 Spectrophotometer (NanoDrop Technologies, Wilmington, DE, USA). To perform the TaqMan allelic discrimination assay with a LightCycler 480 (Roche Diagnostics, Basel, Switzerland), TaqMan SNP Genotyping Assays (Life Technologies) that contained sequence-specific forward and reverse primers to amplify the polymorphic sequence and two probes labeled with VIC and FAM dye to detect both alleles of the rs2653349 and rs726016 SNPs (Assay ID: C___1507491_10 and C__2669260_10, respectively) were used. Real-time polymerase chain reaction (PCR) was performed in a final volume of 10 μl that contained 2×LightCycler 480 Probes Master (Roche Diagnostics), 40×TaqMan Genotyping Assays, 5-50 ng genomic DNA as the template, and up to 10 μl H_2_O equipped with 2×LightCycler 480 Probes Master. The thermal conditions were the following: 95°C for 10 min, followed by 45 cycles of 95°C for 10 s and 60°C for 60 s, with final cooling at 50°C for 30 s. Afterward, endpoint fluorescence was measured for each sample well, and each genotype was determined based on the presence or absence of each type of fluorescence.

Array-based genotyping procedure for data extraction of a specific SNP

To genotype the rs2653349 SNP for a total of 311 DNA samples from healthy subjects with schizotypal personality trait data, array-based genotyping was conducted, and genotype data for this SNP were extracted from the overall results. Briefly, genotyping was performed using Affymetrix Genome-Wide Human SNP Array 6.0 (Affymetrix, Santa Clara, CA, USA) as previously described (19) after venous blood was collected from the subjects and genomic DNA was extracted from whole blood according to standard procedures. The rs2653349 SNP was extracted from this dataset. No deviation from the HWE in the examined SNP was detected (*p* > 0.05; Additional file 14: Table S12).

To genotype the rs2653349 SNP for a total of 2,305 DNA samples from autopsy specimens, array-based genotyping was conducted, and genotype data for this SNP were extracted from the overall results. Briefly, genomic DNA was extracted from the renal cortex using a standard procedure. All of the samples were genotyped using Illumina Infinium HumanExome Beadchips (Illumina, San Diego, CA, USA) with iScan in accordance with the Illumina protocols of the Center for Molecular Biology and Genetics / Core–Lab, Graduate School of Regional Innovation Studies, Mie University, Mie, Japan. Genotype calling was performed on all of the samples as a single project using Genotyping Module 4 (version 1.9) of the Genome Studio Data Analysis software package. Initial genotype clustering was performed using the default Illumina cluster file (Human Exome–12v1–1_A.egt) and manifest file (Human Exome–12v1–1_A.bpm) using the GenTrain2 clustering algorithm.

The characteristics of the study population of these samples are shown in Additional file 10: Table S9. A total of 2,305 (99.4%) of 2,320 subjects were successfully genotyped, among which 2,291 subjects were considered for the demographic statistics and association analysis (Additional file 10: Table S9). Structural alterations in coding SNPs can lead to amino-acid substitutions in the resultant protein encoded by the genome. After the quality control-based deletion of SNPs according to relevant criteria, such as occurrence in or residing on the sex chromosome or HWE with *p* < 10^-6^ in controls, the SNPs were retained for further work-up and final reporting. Only the SNPs with a NoCall rate less than 0.05 and minor allele frequency greater than 0.03 were included in the analyses.

**Statistical analysis**

*Genome-wide association study*

As an initial exploratory study to narrow down candidate SNPs, a two-stage GWAS was conducted for patients with smoking behavior data to investigate the association between genetic variations and the severity of nicotine dependence. Approximately half of the subjects lacked smoking behavior data related to nicotine dependence among 300 subjects who were genotyped. Therefore, a total of 148 subjects were used for our two-stage GWAS (68 and 80 subjects for the first- and second-stage analyses, respectively). In our two-stage GWAS, 225,591 SNPs were selected for the analyses after the filtration step.

As an index of nicotine dependence, the FTND, TDS, and CPD were used for the GWAS. Prior to the analyses, the quantitative values of these measures were classified into two categories by considering the medians of each distribution. The subjects were then divided into two subgroups: low dependence (FTND < 4, TDS < 3, CPD ≤ 20) and high dependence (FTND ≥ 4, TDS ≥ 3, CPD > 20). To explore the association between the SNPs and phenotype, Cochran-Armitage trend tests were conducted in each stage of the analysis, and associations between the phenotypes and genotype distributions were investigated. The association study included both female and male subjects for autosomal markers, although male genotypes were excluded from the analysis of X chromosome markers. All of the statistical analyses were performed using gPLINK v. 2.050, PLINK v. 1.07 (http://pngu.mgh.harvard.edu/purcell/plink/; accessed April 20, 2014) (20), and Haploview v. 4.1 (21).

The GWAS procedure is summarized in Additional file 13: Figure S1. In the first-stage analysis of 68 subjects with smoking behavior data related to nicotine dependence, the SNPs that showed statistical *p* values < 0.05 were selected as candidate SNPs for the second-stage analysis among the 225,591 SNPs. For these SNPs, the second-stage analysis was conducted for 80 subjects with smoking behavior data related to nicotine dependence. Again, SNPs that showed *p* < 0.05 for the single analysis of this stage and combined analysis of the first and second stages were considered potent candidates and selected SNPs for further replication analysis were selected from among these SNPs. In addition, linkage disequilibrium (LD)-based SNP pruning was also conducted in this stage utilizing PLINK v. 1.07 software to remove redundant SNPs due to the strong LD with each other, and SNPs that were in approximate linkage equilibrium with an SNP were excluded based on the following process: (i) consider a window of 50 SNPs, (ii) calculate LD between each pair of SNPs in the window, (iii) remove one of a pair of SNPs if the LD is greater than the *r^2^* value 0.8, and (iv) shift the window five SNPs forward and repeat the procedure. In the confirmatory replication study after the GWAS for 148 subjects, an association analysis was conducted to examine whether the possible association between the SNPs that were selected after the second stage and phenotypic trait would be strictly replicated using samples for the remaining 374 patients among a total of 522 patients who visited Iwata City Hospital and provided smoking behavior data. In this stage, the *q* values of the false discovery rate were calculated to correct for multiple testing, in addition to calculating *p* values, according to previous reports (22, 23). The SNPs that showed *q* < 0.05 in the analysis were considered statistically significant in the present study.

A log quantile-quantile (QQ) *p*-value plot as a result of the GWAS for the combined samples was subsequently drawn to check the pattern of the generated *p*-value distribution, in which the observed *p* values against the values expected from the null hypothesis of uniform distribution, calculated as –log10 (*p* value), were plotted for each model. All of the plots were mostly concordant with the expected line (y = x), especially over the range of 0 < –log10 (*p* value) < 4, indicating no apparent population stratification of the samples used in the study (Additional file 15: Figure S2).

*Association study for candidate SNPs*

Additional analyses were conducted to explore further possible associations between the rs2653349 SNPs and several phenotypic traits, including disease states after the multistage GWAS in the subjects with smoking behavior data. The samples included in these analyses were from the patients who underwent major abdominal surgery, patients with METH dependence/psychosis, and healthy subjects with schizotypal personality trait data. Postmortem samples from autopsy specimens were also evaluated. For all of the genotype data used in these analyses, the distributions were checked for goodness of fit using the *χ^2^* test, and the absence of significant deviation from the theoretical distribution expected from HWE was confirmed (Additional file 14: Table S12).

In the analysis of the patients who underwent major abdominal surgery, NRS pain scores, the frequency of rescue analgesic administration, and the calculated total dose of rescue analgesics administered during the first 24-h postoperative period were used. To explore the association between the rs2653349 SNP and phenotype, the Mann-Whitney *U*-test was performed. For these analyses, the phenotype and genotype data of the SNP were incorporated as dependent and independent variables, respectively. Because we did not find any significant associations between NRS pain score and diagnosis, type of surgery, age, and sex in our preliminary study (data not shown), we did not consider these factors as confounding factors.

Fisher’s exact tests were performed to investigate the contribution of the rs2653349 SNP to the presence of several diseases or several phenotypic traits in the autopsy samples. These analyses were conducted using Cochran-Armitage trend tests by comparing the genotype distributions between the patients and control subjects or between subcategories in the subjects who presented the presence or absence of or tendency toward specific traits.

In the analysis of the healthy subjects with schizotypal personality trait data, differences in clinical characteristics between the genotype groups were analyzed using the *χ^2^* test for categorical variables and Mann-Whitney *U*-test for continuous variables. The effects of the *HCRTR2* genotype on the total score and on the three factors of the SPQ were analyzed using one-way analysis of covariance (ANCOVA). To control confounding factors, age, sex, and years of education were used as covariates because the SPQ total score and three factors were correlated with these confounding factors (24).

For the statistical analyses, SPSS 18.0J for Windows (International Business Machines, Armonk, NY, USA), SNPAlyze v5.1.1 Pro software (Dynacom, Yokohama, Japan), gPLINK v. 2.050, PLINK v. 1.07 (http://pngu.mgh.harvard.edu/purcell/plink/; accessed April 20, 2014) (20), and Haploview v. 4.1 (21) were used, with the exception for the samples from autopsy specimens. The statistical analysis was conducted for samples from autopsy specimens using JMP Genomics software (SAS Institute, Cary, NC, USA). All of the *p* values are two-tailed, and statistical significance was defined as *p* < 0.05, with the exception of the GWAS. Statistical corrections for multiple tests, such as Bonferroni adjustments on the multiple parameters analyzed, were not performed in the present additional exploratory analyses because it would be too conservative for genetic association studies (25), meaning that the likelihood of type II errors is increased by Bonferroni adjustments, and truly important differences would be deemed nonsignificant (26).

**Additional *in silico* analysis**

*Reference to databases*

To scrutinize the candidate SNPs and genes that are possibly associated with the severity of nicotine dependence in humans, we referred to several databases, including the dbSNP database (http://www.ncbi.nlm.nih.gov/snp/; accessed July 16, 2014) and HapMap database (http://hapmap.ncbi.nlm.nih.gov/index.html.ja; accessed July 16, 2015) (27). A PolyPhen-2 (Polymorphism Phenotyping v2) search (http://genetics.bwh.harvard.edu/pph2/index.shtml; accessed April 20, 2014), which is a tool that predicts the possible impact of an amino-acid substitution on the structure and function of a human protein using straightforward physical and comparative considerations (28), was utilized to estimate the functional impact of the candidate SNP, rs2653349. Another web server, SNPinfo Web Server (http://snpinfo.niehs.nih.gov/; accessed July 16, 2015) (29), was utilized to obtain functional information.

*Power analysis*

Statistical power analyses were preliminarily performed using G*Power version 3.0.5 (30). Power analyses for the *χ^2^* tests, with the degrees of freedom set at 2, revealed that the expected power (1 minus type II error probability) was 96.7% and 98.5% for Cohen’s conventional “large” effect size of 0.5 (31) when the type I error probability was set at 0.05 and the sample sizes were 68 and 80, respectively, corresponding to the sample sizes of the first- and second-stage analyses in the present study. However, for the same type I error probability and sample sizes of 68 and 80, the expected power decreased to 59.3% and 66.9%, respectively, when Cohen’s conventional “medium” effect size was 0.3. Conversely, the estimated effect sizes were 0.3764 and 0.3470 for the same type I error probability and sample sizes of 68 and 80, respectively, to achieve 80% power. Therefore, a single analysis in the present study was expected to detect true associations with the phenotype with 80% statistical power for effect sizes from large to moderately medium but not small.

*Imputation analysis*

For the fine mapping of SNPs that are close to the candidate locus and possibly associated with nicotine dependence, imputation analysis was conducted to extrapolate the missing genotype data between our data that were obtained from whole-genome genotyping after the quality control process. The analysis was performed using the “--proxy-impute all” and several related commands within gPLINK v. 2.050 and v. PLINK 1.07 software (20), based on the whole-genome genotyping data and local genotype data of the Japanese and Han Chinese populations (release #23) extracted from the 300 kbp region that encompasses the rs2653349 SNP, which is located at 55,250,296 on chromosome 6 in the HapMap database (27). After the imputation analysis, an additional association study was conducted as mentioned above based on the imputed genotype data. The results for SNPs that met the criterion *p* > 0.001 in the HWE test were used to depict a plot for fine mapping.

**References**

1. Sato N, Kageyama S, Chen R, Suzuki M, Mori H, Tanioka F *et al.* Association between neuropeptide Y receptor 2 polymorphism and the smoking behavior of elderly Japanese. *J Hum Genet* 2010; **55**(11): 755-760.

2. Ella E, Sato N, Nishizawa D, Kageyama S, Yamada H, Kurabe N *et al.* Association between dopamine beta hydroxylase rs5320 polymorphism and smoking behaviour in elderly Japanese. *J Hum Genet* 2012; **57**(6): 385-390.

3. Heatherton TF, Kozlowski LT, Frecker RC, Fagerstrom KO. The Fagerstrom Test for Nicotine Dependence: a revision of the Fagerstrom Tolerance Questionnaire. *Br J Addict* 1991; **86**(9): 1119-1127.

4. Kawakami N, Takatsuka N, Inaba S, Shimizu H. Development of a screening questionnaire for tobacco/nicotine dependence according to ICD-10, DSM-III-R, and DSM-IV. *Addict Behav* 1999; **24**(2): 155-166.

5. Hayashida M, Nagashima M, Satoh Y, Katoh R, Tagami M, Ide S *et al.* Analgesic requirements after major abdominal surgery are associated with *OPRM1* gene polymorphism genotype and haplotype. *Pharmacogenomics* 2008; **9**(11): 1605-1616.

6. Nishizawa D, Nagashima M, Katoh R, Satoh Y, Tagami M, Kasai S *et al.* Association between *KCNJ6* (*GIRK2*) gene polymorphisms and postoperative analgesic requirements after major abdominal surgery. *PLoS One* 2009; **4**(9): e7060.

7. Ujike H, Harano M, Inada T, Yamada M, Komiyama T, Sekine Y *et al.* Nine- or fewer repeat alleles in VNTR polymorphism of the dopamine transporter gene is a strong risk factor for prolonged methamphetamine psychosis. *Pharmacogenomics J* 2003; **3**(4): 242-247.

8. Ujike H. [Japanese Genetics Initiative for Drug Abuse (JGIDA)]. *Nihon Shinkei Seishin Yakurigaku Zasshi* 2004; **24**(5): 299-302.

9. Ujike H, Kishimoto M, Okahisa Y, Kodama M, Takaki M, Inada T *et al.* Association between 5HT1b receptor gene and methamphetamine dependence. *Curr Neuropharmacol* 2011; **9**(1): 163-168.

10. Kishi T, Kitajima T, Kawashima K, Okochi T, Yamanouchi Y, Kinoshita Y *et al.* Association analysis of nuclear receptor Rev-erb alpha gene (*NR1D1*) and Japanese methamphetamine dependence. *Curr Neuropharmacol* 2011; **9**(1): 129-132.

11. Kishi T, Kitajima T, Tsunoka T, Okumura T, Kawashima K, Okochi T *et al.* Lack of association between prokineticin 2 gene and Japanese methamphetamine dependence. *Curr Neuropharmacol* 2011; **9**(1): 133-136.

12. Iijima Y, Sasaki J, Bando N, Asai T, Mouri I, Tanno Y. Development of a Japanese version of the Schizotypal Personality Questionnaire and factor structure of schizotypy. *Koudouryouhoukenkyu* 2010; **36**: 29-41.

13. Someya T, Sasaki T, Takahashi S. Reliability and validity of schizotypal personality questionnaire. *Proceeding of the 32nd Scientific Meeting of the University Health Care in Japan* 1994: 286-290.

14. Raine A. The SPQ: a scale for the assessment of schizotypal personality based on DSM-III-R criteria. *Schizophr Bull* 1991; **17**(4): 555-564.

15. Raine A, Reynolds C, Lencz T, Scerbo A, Triphon N, Kim D. Cognitive-perceptual, interpersonal, and disorganized features of schizotypal personality. *Schizophr Bull* 1994; **20**(1): 191-201.

16. Wechsler D. The Manual for the Wechsler Intelligence Scale - Third Edition (WISC-III). The Psychological Corporation: San Antonio, TX, 1991.

17. Sawabe M, Arai T, Kasahara I, Esaki Y, Nakahara K, Hosoi T *et al.* Developments of geriatric autopsy database and Internet-based database of Japanese single nucleotide polymorphisms for geriatric research (JG-SNP). *Mech Ageing Dev* 2004; **125**(8): 547-552.

18. Nishizawa D, Hayashida M, Nagashima M, Koga H, Ikeda K. Genetic polymorphisms and human sensitivity to opioid analgesics. *Methods Mol Biol* 2010; **617**: 395-420.

19. Hashimoto R, Ikeda M, Ohi K, Yasuda Y, Yamamori H, Fukumoto M *et al.* Genome-wide association study of cognitive decline in schizophrenia. *Am J Psychiatry* 2013; **170**(6): 683-684.

20. Purcell S, Neale B, Todd-Brown K, Thomas L, Ferreira MA, Bender D *et al.* PLINK: a tool set for whole-genome association and population-based linkage analyses. *Am J Hum Genet* 2007; **81**(3): 559-575.

21. Barrett JC, Fry B, Maller J, Daly MJ. Haploview: analysis and visualization of LD and haplotype maps. *Bioinformatics* 2005; **25**(2): 263-265.

22. Benjamini Y, Hochberg Y. Controlling the false discovery rate: a practical and powerful approach to multiple testing. *J R Statist Soc B* 1995; **57**: 289-300.

23. Storey J. The positive False Discovery Rate: a Bayesian interpretation and the q-value. *Ann Statist* 2003; **31**(6): 2013-2035.

24. Ma X, Sun J, Yao J, Wang Q, Hu X, Deng W *et al.* A quantitative association study between schizotypal traits and COMT, PRODH and BDNF genes in a healthy Chinese population. *Psychiatry Res* 2007; **153**(1): 7-15.

25. Nyholt DR. Genetic case-control association studies--correcting for multiple testing. *Hum Genet* 2001; **109**(5): 564-567.

26. Perneger TV*.* What's wrong with Bonferroni adjustments. *BMJ* 1998; **316**(7139): 1236-1238.

27. International HapMap Consortium*.* A haplotype map of the human genome. *Nature* 2005; **437**(7063): 1299-1320.

28. Adzhubei IA, Schmidt S, Peshkin L, Ramensky VE, Gerasimova A, Bork P *et al.* A method and server for predicting damaging missense mutations. *Nat Methods* 2010; **7**(4): 248-249.

29. Xu Z, Taylor JA*.* SNPinfo: integrating GWAS and candidate gene information into functional SNP selection for genetic association studies. *Nucleic Acids Res* 2009; **37**: W600-W605.

30. Faul F, Erdfelder E, Lang AG, Buchner A. G*Power 3: a flexible statistical power analysis program for the social, behavioral, and biomedical sciences. *Behav Res Methods* 2007; **39**(2): 175-191.

31. Cohen J. *Statistical power analysis for the behavioral sciences*, revised edition. New York: Academic Press, 1977.
