# Supplementary material for: Associations between the orexin (hypocretin) receptor 2 gene polymorphism Val308Ile and nicotine dependence in genome-wide and subsequent association studies
Source: Mol Brain. 2015 Aug 20;8:50. doi: 10.1186/s13041-015-0142-x (PMC4546081; doi:10.1186/s13041-015-0142-x)
Supplement: Additional file 6: Table S5. — Demographic and clinical data of the subjects who underwent major abdominal surgery. (DOC 74 kb) [file 13041_2015_142_MOESM6_ESM.doc]

| **Table S5. Demographic and clinical data of the subjects who underwent major abdominal surgery.** | | | | | | | | |
| --- | --- | --- | --- | --- | --- | --- | --- | --- |
|  |  |  |  |  |  |  |  |  |
|  |  | ***n*** | **Minimum** | **Maximum** | **Mean** | **SD** | **Median** | ***p*** |
|  |  |  |  |  |  |  |  |  |
| **Gender** |  |  |  |  |  |  |  |  |
| male |  | 60 |  |  |  |  |  |  |
| female |  | 52 |  |  |  |  |  |  |
|  |  |  |  |  |  |  |  |  |
| **Age** |  | 112 | 28 | 80 | 63.13 | 10.06 | 63.00 |  |
|  |  |  |  |  |  |  |  |  |
| **Height (cm)** |  | 112 | 133 | 175 | 157.89 | 8.00 | 158.00 |  |
|  |  |  |  |  |  |  |  |  |
| **Weight (kg)** |  | 112 | 38 | 77 | 55.83 | 9.97 | 54.00 |  |
|  |  |  |  |  |  |  |  |  |
| **NRS pain score** |  | 90 | 0 | 4 | 1.51 | 1.25 | 1 |  |
| rs2653349 A/G genotype |  | 12 | 0 | 2 | 0.5 | 0.67 | 0 | 0.0017* |
| rs2653349 G/G genotype |  | 78 | 0 | 4 | 1.67 | 1.24 | 1 |  |
|  |  |  |  |  |  |  |  |  |
| **Frequency of analgesic administration** |  | 112 | 0 | 6 | 0.72 | 1.00 | 0 |  |
| rs2653349 A/G genotype |  | 13 | 0 | 3 | 0.46 | 0.97 | 0 | 0.3905 |
| rs2653349 G/G genotype |  | 99 | 0 | 6 | 0.76 | 1.00 | 1 |  |
|  |  |  |  |  |  |  |  |  |
| **Total dose of rescue analgesic (μg/kg)** |  | 112 | 0.00 | 6.48 | 0.77 | 1.15 | 0.00 |  |
| rs2653349 A/G genotype |  | 13 | 0.00 | 2.17 | 0.37 | 0.76 | 0.00 | 0.1091 |
| rs2653349 G/G genotype |  | 99 | 0.00 | 6.48 | 0.82 | 1.19 | 0.41 |  |
|  |  |  |  |  |  |  |  |  |
|  |  |  |  |  |  |  |  |  |
| *n*, number of samples; *, significantly less pain for the A/G genotype compared with the G/G genotype. | | | | | | | | |
