# Supplementary material for: Associations between the orexin (hypocretin) receptor 2 gene polymorphism Val308Ile and nicotine dependence in genome-wide and subsequent association studies
Source: Mol Brain. 2015 Aug 20;8:50. doi: 10.1186/s13041-015-0142-x (PMC4546081; doi:10.1186/s13041-015-0142-x)
Supplement: Additional file 7: Table S6. — Demographic and clinical data of patient subjects with methamphetamine dependence/psychosis. (DOC 72 kb) [file 13041_2015_142_MOESM7_ESM.doc]

| **Table S6. Demographic and clinical data of patient subjects with methamphetamine dependence/psychosis.** | | | | | | | | | |
| --- | --- | --- | --- | --- | --- | --- | --- | --- | --- |
|  |  |  |  |  |  |  |  |  |  |
|  |  | ***n*** | **Minimum** | **Maximum** | **Mean** | **SD** | **Median** | ***p*** |  |
|  |  |  |  |  |  |  |  |  |  |
| **Gender of all patients with dependence** |  |  |  |  |  |  |  |  |  |
| male |  | 165 |  |  |  |  |  |  |  |
| female |  | 38 |  |  |  |  |  |  |  |
|  |  |  |  |  |  |  |  |  |  |
| **Gender of patients with comorbid psychosis** |  |  |  |  |  |  |  |  |  |
| male |  | 155 |  |  |  |  |  |  |  |
| female |  | 30 |  |  |  |  |  |  |  |
|  |  |  |  |  |  |  |  |  |  |
| **Age (years)** |  | 202 | 18 | 69 | 37.82 | 12.05 | 35.00 |  |  |
|  |  |  |  |  |  |  |  |  |  |
| **First use of methamphetamine (years)** |  | 200 | 13 | 46 | 20.78 | 5.49 | 19 |  |  |
| rs2653349 A/G genotype |  | 94 | 13 | 28 | 18.20 | 3.90 | 17 | 0.0056* |  |
| rs2653349 G/G genotype |  | 84 | 13 | 46 | 21.05 | 5.58 | 19 |  |  |
|  |  |  |  |  |  |  |  |  |  |
| **Number of drugs used** |  | 186 | 1 | 10 | 3.12 | 1.71 | 2 |  |  |
| rs2653349 A/G genotype |  | 94 | 1 | 5 | 2.60 | 1.19 | 2 | 0.2601 |  |
| rs2653349 G/G genotype |  | 84 | 1 | 10 | 2.55 | 1.80 | 2 |  |  |
|  |  |  |  |  |  |  |  |  |  |
|  |  |  |  |  |  |  |  |  |  |
| *n*, number of samples; *, significantly earlier first use of methamphetamine for the A/G genotype compared with the G/G genotype. | | | | | | | | | |
