# Supplementary material for: Associations between the orexin (hypocretin) receptor 2 gene polymorphism Val308Ile and nicotine dependence in genome-wide and subsequent association studies
Source: Mol Brain. 2015 Aug 20;8:50. doi: 10.1186/s13041-015-0142-x (PMC4546081; doi:10.1186/s13041-015-0142-x)
Supplement: Additional file 8: Table S7. — Demographic variables for subjects included in the SPQ analysis (mean ± SD). (DOC 52 kb) [file 13041_2015_142_MOESM8_ESM.doc]

| **Table S7.** Demographic variables for subjects included in the SPQ analysis (mean ± SD). | | | | | | | |
| --- | --- | --- | --- | --- | --- | --- | --- |
|  |  |  |  |  |  |  |  |
|  | **Total** |  | **A/G** | **G/G** |  | ***p*** | **(*z*)** |
| **Variable** | **(*n* = 311)** |  | **(*n* = 24)** | **(*n* = 287)** |  |  |  |
| Age (years) | 36.4 ± 12.4 |  | 31.0 ± 9.8 | 36.9 ± 12.5 |  | **0.047** | 1.99 |
| Sex (male/female) ^a^ | 129/182 |  | 7/17 | 122/165 |  | 0.2 | 1.62 |
| Education (years) | 14.8 ± 2.3 |  | 16.4 ± 1.7 | 14.8 ± 2.3 |  | **< 0.001** | -3.74 |
| Full-scale IQ | 109.0 ± 12.3 |  | 113.8 ± 11.5 | 108.6 ± 12.3 |  | 0.063 | -1.86 |
| SPQ, Schizotypal Personality Questionnaire. Value of *p* < 0.05 are in bold. ^a^ *χ*^2^ test. | | | | | | | |
