# Supplementary material for: Associations between the orexin (hypocretin) receptor 2 gene polymorphism Val308Ile and nicotine dependence in genome-wide and subsequent association studies
Source: Mol Brain. 2015 Aug 20;8:50. doi: 10.1186/s13041-015-0142-x (PMC4546081; doi:10.1186/s13041-015-0142-x)
Supplement: Additional file 9: Table S8. — Impact of the HCRTR2 gene risk variant on schizotypal personality traits (mean ± SD). (DOC 54 kb) [file 13041_2015_142_MOESM9_ESM.doc]

| **Table S8. Impact of the *HCRTR2* gene risk variant on schizotypal personality traits (mean ± SD).** | | | | | | | |
| --- | --- | --- | --- | --- | --- | --- | --- |
|  |  |  |  |  |  |  |  |
| **SPQ** | **Total** |  | **A/G** | **G/G** |  | **Genotype effect** | |
| **Variable** | **(*n* = 311)** |  | **(*n* = 24)** | **(*n* = 287)** |  | ***F*_1,306_** | ***p*** |
| Total score | 12.3 ± 10.1 |  | 16.6 ± 14.7 | 12.0 ± 9.6 |  | 5.8 | **0.017** |
|  |  |  |  |  |  |  |  |
| Cognitive/perceptual | 3.9 ± 4.5 |  | 5.5 ± 6.9 | 3.8 ± 4.2 |  | 4.37 | **0.037** |
| Interpersonal | 6.0 ± 5.2 |  | 7.4 ± 6.8 | 5.8 ± 5.1 |  | 2.89 | 0.09 |
| Disorganization | 3.5 ± 3.5 |  | 4.8 ± 4.6 | 3.3 ± 3.4 |  | 4.19 | **0.042** |
| SPQ, Schizotypal Personality Questionnaire. Significant *p* values are shown in bold. | | | | | |  |  |
