# Supplementary material for: Associations between the orexin (hypocretin) receptor 2 gene polymorphism Val308Ile and nicotine dependence in genome-wide and subsequent association studies
Source: Mol Brain. 2015 Aug 20;8:50. doi: 10.1186/s13041-015-0142-x (PMC4546081; doi:10.1186/s13041-015-0142-x)
Supplement: Additional file 10: Table S9. — Demographic and clinical data of patient subjects with autopsy specimens. (DOC 139 kb) [file 13041_2015_142_MOESM10_ESM.doc]

| **Table S9. Demographic and clinical data of patient subjects with autopsy specimens.** | | | | | | | | | | |
| --- | --- | --- | --- | --- | --- | --- | --- | --- | --- | --- |
|  |  |  |  |  |  |  |  |  |  |  |
| **Demographic data** | | ***n*** | **Minimum** | **Maximum** | **Mean** | **SD** | **Median** |  |  |  |
|  |  |  |  |  |  |  |  |  |  |  |
| **Gender of all patients** | |  |  |  |  |  |  |  |  |  |
| male |  | 1256 |  |  |  |  |  |  |  |  |
| female |  | 1021 |  |  |  |  |  |  |  |  |
| uncertain |  | 14 |  |  |  |  |  |  |  |  |
|  |  |  |  |  |  |  |  |  |  |  |
| **Age (years)** |  | 2290 | 33 | 104 | 80.59 | 8.79 | 81 |  |  |  |
|  |  |  |  |  |  |  |  |  |  |  |
| **Drinking/Smoking status** | | **absence** | **presence** |  |  |  |  |  |  |  |
|  |  |  |  |  |  |  |  |  |  |  |
| drinking |  | 810 | 473 |  |  |  |  |  |  |  |
| smoking |  | 1035 | 1122 |  |  |  |  |  |  |  |
| heavy smoking (1)^†^ | | 1826 | 276 |  |  |  |  |  |  |  |
| heavy smoking (2)^§^ | | 750 | 257 |  |  |  |  |  |  |  |
|  |  |  |  |  |  |  |  |  |  |  |
| **Clinical data (disease status):** | | **absence** | **presence** |  | **Clinical data (disease status):** | |  |  | **absence** | **presence** |
|  |  |  |  |  |  |  |  |  |  |  |
| acute leukemia | | 1614 | 89 |  | liver cancer |  |  |  | 1645 | 61 |
| Alzheimer’s disease | | 1160 | 225 |  | lung adenocarcinoma | |  |  | 2166 | 118 |
| amyotrophic lateral sclerosis | | 2239 | 24 |  | lung cancer |  |  |  | 2022 | 262 |
| aneurysm |  | 1573 | 130 |  | lung squamous adenocarcinoma | | |  | 2276 | 8 |
| aortic aneurysm | | 1639 | 69 |  | lung thromboembolism | |  |  | 1592 | 111 |
| aortic dissection | | 1670 | 33 |  | lung tuberculosis | |  |  | 1434 | 269 |
| aortic valve calcification | | 1604 | 100 |  | lymphocytic leukemia | |  |  | 2265 | 20 |
| arteriosclerosis obliterans | | 1643 | 65 |  | malignant hematopoietic neoplasm | | |  | 1470 | 238 |
| aspiration |  | 1592 | 116 |  | malignant lymphoma | |  |  | 2159 | 125 |
| atrial fibrillation | | 1493 | 215 |  | malnutrition |  |  |  | 1393 | 310 |
| biliary tract cancer | | 2223 | 64 |  | melanoma |  |  |  | 2282 | 2 |
| blood cancer |  | 2076 | 209 |  | mesothelioma |  |  |  | 2284 | 1 |
| brain hemorrhage | | 1228 | 158 |  | mitral valve calcification | |  |  | 1629 | 75 |
| brain infarction | | 728 | 661 |  | myelodysplastic syndrome | |  |  | 2244 | 41 |
| brain tumor |  | 2283 | 2 |  | myelogenous leukemia | |  |  | 2173 | 112 |
| breast cancer |  | 2203 | 81 |  | myeloma |  |  |  | 2245 | 40 |
| cancer of small intestine | | 2276 | 11 |  | myocardial infarction | |  |  | 1321 | 383 |
| cataracts |  | 1587 | 121 |  | osteoarthritis deformans | |  |  | 1645 | 63 |
| cerebral infarction | | 740 | 636 |  | osteoporosis |  |  |  | 1490 | 213 |
| cerebrovascular disease | | 1214 | 494 |  | other tumors |  |  |  | 2274 | 10 |
| cholelithiasis |  | 1459 | 247 |  | ovarian cancer | |  |  | 1020 | 4 |
| chronic hepatitis / liver cirrhosis | | 1579 | 127 |  | pancreatic cancer | |  |  | 2218 | 67 |
| colon cancer |  | 2120 | 167 |  | Parkinson’s disease | |  |  | 1325 | 60 |
| colorectal cancer | | 1550 | 156 |  | pneumonia |  |  |  | 850 | 853 |
| COPD (chronic obstructive | | 1252 | 147 |  | presence of clinical cancer | |  |  | 993 | 427 |
| pulmonary disease) | |  |  |  | presence of latent cancer | |  |  | 1214 | 205 |
| decubitus ulcer | | 1664 | 44 |  | prostate hypertrophy | |  |  | 1603 | 105 |
| degenerative valvular disease | | 1589 | 115 |  | prostate cancer | |  |  | 1054 | 210 |
| dementia |  | 1483 | 225 |  | pyelitis and pyelonephritis | |  |  | 1540 | 166 |
| diabetes |  | 1193 | 206 |  | rectal cancer | |  |  | 2232 | 55 |
| diabetic nephropathy | | 1625 | 81 |  | reflux esophagitis | |  |  | 1549 | 154 |
| diverticulosis |  | 1513 | 193 |  | rheumatoid arthritis (RA) | |  |  | 2189 | 74 |
| duodenal ulcer | | 1632 | 74 |  | sarcoma |  |  |  | 2277 | 7 |
| esophageal cancer | | 2252 | 31 |  | sepsis |  |  |  | 1215 | 204 |
| femoral fracture | | 2070 | 193 |  | skin cancer |  |  |  | 2272 | 9 |
| gallbladder and bile duct cancer | | 1652 | 54 |  | small-cell carcinoma of the lung | |  |  | 2225 | 59 |
| gastric cancer |  | 2023 | 264 |  | smoking-related emphysema | |  |  | 704 | 185 |
| gastric ulcer |  | 1407 | 299 |  | squamous-cell carcinoma of the lung | | |  | 2204 | 80 |
| glaucoma |  | 1691 | 17 |  | thyroid cancer |  |  |  | 2230 | 54 |
| goiter |  | 1662 | 41 |  | thyroiditis |  |  |  | 1647 | 56 |
| head and neck cancer | | 2261 | 23 |  | total cancer presence | |  |  | 870 | 1419 |
| hepatocellular carcinoma | | 2216 | 71 |  | tuberculosis |  |  |  | 1403 | 300 |
| hyperlipidemia |  | 1368 | 31 |  | type 2 diabetes | |  |  | 1451 | 257 |
| hypertension |  | 1205 | 503 |  | unclassified lung cancer | |  |  | 2274 | 10 |
| idiopathic interstitial pneumonia | | 1677 | 31 |  | unclassified tumor | |  |  | 2281 | 3 |
| interstitial pneumonia | | 1590 | 113 |  | urinary tract cancer | |  |  | 2241 | 46 |
| ischemic colon disease | | 1567 | 139 |  | urinary tract infection | |  |  | 1645 | 63 |
| ischemic heart disease | | 1437 | 271 |  | urolithiasis |  |  |  | 1658 | 48 |
| kidney cancer |  | 2253 | 34 |  | uterine cancer |  |  |  | 998 | 21 |
| large-cell carcinoma of the lung | | 2280 | 4 |  | vertebral fracture | |  |  | 2080 | 183 |
| left ventricular hypertrophy | | 1230 | 474 |  |  |  |  |  |  |  |
|  |  |  |  |  |  |  |  |  |  |  |
|  |  |  |  |  |  |  |  |  |  |  |
| ^†^, absence/presence of heavy-smoking in smokers and non-smokers; ^§^, absence/presence of heavy-smoking in smokers. | | | | | | | |  |  |  |
