# Supplementary material for: Associations between the orexin (hypocretin) receptor 2 gene polymorphism Val308Ile and nicotine dependence in genome-wide and subsequent association studies
Source: Mol Brain. 2015 Aug 20;8:50. doi: 10.1186/s13041-015-0142-x (PMC4546081; doi:10.1186/s13041-015-0142-x)
Supplement: Additional file 11: Table S10. — Results of analysis of associations between the rs2653349 SNP and diseases/phenotypes. (DOC 221 kb) [file 13041_2015_142_MOESM11_ESM.doc]

| **Table S10. Results of analysis of associations between the rs2653349 SNP and diseases/phenotypes.** | | | | | | | | | | | | |
| --- | --- | --- | --- | --- | --- | --- | --- | --- | --- | --- | --- | --- |
|  |  |  |  |  |  |  |  |  |  |  |  |  |
| **Rank** |  | **Disease (Phenotype)** |  | ***p*** |  | **Genotype (Control)** | | |  | **Genotype (Case)** | | |
|  |  |  |  |  |  | **A/A** | **A/G** | **G/G** |  | **A/A** | **A/G** | **G/G** |
|  |  |  |  |  |  |  |  |  |  |  |  |  |
| 1 |  | goiter |  | 1.573E-05* |  | 3 | 138 | 1517 |  | 0 | 12 | 30 |
| 2 |  | aortic aneurysm |  | 0.0176* |  | 3 | 138 | 1495 |  | 0 | 12 | 57 |
| 3 |  | myeloma |  | 0.0236* |  | 3 | 202 | 2038 |  | 0 | 8 | 32 |
| 4 |  | amyotrophic lateral sclerosis |  | 0.0460* |  | 3 | 205 | 2056 |  | 0 | 5 | 18 |
| 5 |  | arteriosclerosis obliterans |  | 0.0816 |  | 2 | 142 | 1496 |  | 1 | 8 | 56 |
| 6 |  | diverticulosis |  | 0.0953 |  | 3 | 126 | 1382 |  | 0 | 24 | 168 |
| 7 |  | mitral valve calcification |  | 0.0985 |  | 3 | 139 | 1484 |  | 0 | 11 | 64 |
| 8 |  | dementia |  | 0.1093 |  | 3 | 136 | 1341 |  | 0 | 14 | 211 |
| 9 |  | drinking |  | 0.1112 |  | 2 | 58 | 758 |  | 1 | 46 | 422 |
| 10 |  | cerebrovascular disease |  | 0.1144 |  | 1 | 100 | 1109 |  | 2 | 50 | 443 |
| 11 |  | atrial fibrillation |  | 0.1168 |  | 2 | 126 | 1362 |  | 1 | 24 | 190 |
| 12 |  | vertebral fracture |  | 0.1215 |  | 3 | 199 | 1906 |  | 0 | 11 | 168 |
| 13 |  | liver cancer |  | 0.1309 |  | 3 | 141 | 1498 |  | 0 | 9 | 52 |
| 14 |  | hepatocellular carcinoma |  | 0.1819 |  | 3 | 200 | 2011 |  | 0 | 10 | 61 |
| 15 |  | idiopathic interstitial pneumonia |  | 0.1827 |  | 3 | 145 | 1526 |  | 0 | 5 | 26 |
| 16 |  | heavy smoking (1)^†^ |  | 0.1852 |  | 3 | 158 | 1633 |  | 0 | 31 | 233 |
| 17 |  | blood cancer |  | 0.1928 |  | 3 | 185 | 1887 |  | 0 | 25 | 183 |
| 18 |  | ischemic heart disease |  | 0.1986 |  | 2 | 133 | 1300 |  | 1 | 17 | 252 |
| 19 |  | hypertension |  | 0.2019 |  | 2 | 99 | 1102 |  | 1 | 51 | 450 |
| 20 |  | aneurysm |  | 0.2075 |  | 2 | 136 | 1432 |  | 1 | 14 | 115 |
| 21 |  | esophageal cancer |  | 0.2091 |  | 3 | 205 | 2042 |  | 0 | 5 | 26 |
| 22 |  | heavy smoking (2)^§^ |  | 0.2099 |  | 1 | 69 | 710 |  | 0 | 31 | 232 |
| 23 |  | malignant lymphoma |  | 0.2356 |  | 3 | 202 | 1952 |  | 0 | 8 | 117 |
| 24 |  | degenerative valvular disease |  | 0.2577 |  | 3 | 136 | 1447 |  | 0 | 14 | 101 |
| 25 |  | myelodysplastic syndrome |  | 0.2606 |  | 3 | 204 | 2035 |  | 0 | 6 | 35 |
| 26 |  | unclassified lung cancer |  | 0.2613 |  | 2 | 210 | 2060 |  | 1 | 0 | 9 |
| 27 |  | ovarian cancer |  | 0.2626 |  | 2 | 85 | 933 |  | 0 | 1 | 3 |
| 28 |  | brain infarction |  | 0.2629 |  | 2 | 56 | 669 |  | 1 | 64 | 593 |
| 29 |  | cerebral infarction |  | 0.2629 |  | 2 | 56 | 669 |  | 1 | 64 | 593 |
| 30 |  | Parkinson’s disease |  | 0.2689 |  | 3 | 117 | 1201 |  | 0 | 3 | 57 |
| 31 |  | small-cell carcinoma of the lung |  | 0.2837 |  | 3 | 202 | 2018 |  | 0 | 8 | 51 |
| 32 |  | cancer of small intestine |  | 0.2900 |  | 3 | 210 | 2061 |  | 0 | 0 | 11 |
| 33 |  | smoking-related emphysema |  | 0.2973 |  | 1 | 80 | 688 |  | 0 | 16 | 181 |
| 34 |  | kidney cancer |  | 0.2988 |  | 3 | 205 | 2043 |  | 0 | 5 | 29 |
| 35 |  | COPD (chronic obstructive pulmonary disease) | | 0.3283 |  | 3 | 100 | 1147 |  | 0 | 16 | 130 |
| 36 |  | rectal cancer |  | 0.3291 |  | 2 | 209 | 2020 |  | 1 | 1 | 52 |
| 37 |  | lung thromboembolism |  | 0.3311 |  | 1 | 141 | 1448 |  | 2 | 9 | 99 |
| 38 |  | skin cancer |  | 0.3380 |  | 3 | 210 | 2057 |  | 0 | 0 | 9 |
| 39 |  | pneumonia |  | 0.3461 |  | 2 | 68 | 777 |  | 1 | 82 | 770 |
| 40 |  | lung squamous adenocarcinoma |  | 0.3668 |  | 3 | 210 | 2061 |  | 0 | 0 | 8 |
| 41 |  | prostate hypertrophy |  | 0.3723 |  | 3 | 143 | 1454 |  | 0 | 7 | 98 |
| 42 |  | reflux esophagitis |  | 0.3821 |  | 3 | 139 | 1405 |  | 0 | 11 | 142 |
| 43 |  | sarcoma |  | 0.3987 |  | 3 | 210 | 2062 |  | 0 | 0 | 7 |
| 44 |  | lung tuberculosis |  | 0.4061 |  | 3 | 129 | 1299 |  | 0 | 21 | 248 |
| 45 |  | rheumatoid arthritis |  | 0.4100 |  | 3 | 205 | 2004 |  | 0 | 5 | 70 |
| 46 |  | urolithiasis |  | 0.4255 |  | 3 | 144 | 1508 |  | 0 | 6 | 42 |
| 47 |  | chronic hepatitis / liver cirrhosis |  | 0.4586 |  | 3 | 136 | 1437 |  | 0 | 14 | 113 |
| 48 |  | lung cancer |  | 0.4794 |  | 2 | 184 | 1834 |  | 1 | 26 | 235 |
| 49 |  | urinary tract cancer |  | 0.4989 |  | 3 | 207 | 2029 |  | 0 | 3 | 43 |
| 50 |  | lymphocytic leukemia |  | 0.5000 |  | 3 | 209 | 2051 |  | 0 | 1 | 19 |
| 51 |  | large-cell carcinoma of the lung |  | 0.5238 |  | 3 | 210 | 2065 |  | 0 | 0 | 4 |
| 52 |  | femoral fracture |  | 0.5313 |  | 1 | 193 | 1897 |  | 2 | 17 | 177 |
| 53 |  | total cancer presence |  | 0.5364 |  | 1 | 76 | 794 |  | 2 | 134 | 1280 |
| 54 |  | pyelitis and pyelonephritis |  | 0.5564 |  | 3 | 137 | 1398 |  | 0 | 13 | 152 |
| 55 |  | unclassified tumor |  | 0.5809 |  | 3 | 210 | 2066 |  | 0 | 0 | 3 |
| 56 |  | breast cancer |  | 0.5845 |  | 3 | 201 | 1998 |  | 0 | 9 | 71 |
| 57 |  | diabetes |  | 0.5867 |  | 2 | 98 | 1091 |  | 1 | 18 | 186 |
| 58 |  | presence of clinical cancer |  | 0.5891 |  | 2 | 87 | 904 |  | 0 | 43 | 383 |
| 59 |  | malnutrition |  | 0.6020 |  | 3 | 124 | 1263 |  | 0 | 26 | 284 |
| 60 |  | thyroid cancer |  | 0.6066 |  | 3 | 206 | 2019 |  | 0 | 4 | 50 |
| 61 |  | decubitus ulcer |  | 0.6132 |  | 3 | 145 | 1513 |  | 0 | 5 | 39 |
| 62 |  | gastric cancer |  | 0.6369 |  | 3 | 183 | 1836 |  | 0 | 27 | 236 |
| 63 |  | prostate cancer |  | 0.6402 |  | 1 | 105 | 947 |  | 0 | 19 | 190 |
| 64 |  | glaucoma |  | 0.6456 |  | 3 | 149 | 1536 |  | 0 | 1 | 16 |
| 65 |  | interstitial pneumonia |  | 0.6510 |  | 3 | 141 | 1443 |  | 0 | 9 | 104 |
| 66 |  | melanoma |  | 0.6523 |  | 3 | 210 | 2067 |  | 0 | 0 | 2 |
| 67 |  | brain tumor |  | 0.6523 |  | 3 | 210 | 2068 |  | 0 | 0 | 2 |
| 68 |  | gallbladder and bile duct cancer |  | 0.6567 |  | 3 | 146 | 1500 |  | 0 | 4 | 50 |
| 69 |  | hyperlipidemia |  | 0.6568 |  | 3 | 114 | 1248 |  | 0 | 2 | 29 |
| 70 |  | thyroiditis |  | 0.6576 |  | 3 | 144 | 1498 |  | 0 | 6 | 49 |
| 71 |  | aortic valve calcification |  | 0.6602 |  | 3 | 142 | 1455 |  | 0 | 8 | 93 |
| 72 |  | smoking |  | 0.6842 |  | 2 | 89 | 924 |  | 1 | 104 | 989 |
| 73 |  | myocardial infarction |  | 0.6846 |  | 1 | 117 | 1202 |  | 2 | 33 | 346 |
| 74 |  | ischemic colon disease |  | 0.7033 |  | 3 | 136 | 1425 |  | 0 | 14 | 125 |
| 75 |  | lung adenocarcinoma |  | 0.7100 |  | 3 | 200 | 1961 |  | 0 | 10 | 108 |
| 76 |  | Alzheimer’s disease |  | 0.7224 |  | 2 | 103 | 1052 |  | 1 | 17 | 206 |
| 77 |  | acute leukemia |  | 0.7313 |  | 3 | 141 | 1468 |  | 0 | 9 | 79 |
| 78 |  | cataracts |  | 0.7315 |  | 3 | 140 | 1441 |  | 0 | 10 | 111 |
| 79 |  | osteoarthritis deformans |  | 0.7389 |  | 3 | 145 | 1494 |  | 0 | 5 | 58 |
| 80 |  | tuberculosis |  | 0.7413 |  | 3 | 124 | 1273 |  | 0 | 26 | 274 |
| 81 |  | mesothelioma |  | 0.7501 |  | 3 | 210 | 2069 |  | 0 | 0 | 1 |
| 82 |  | colorectal cancer |  | 0.7508 |  | 2 | 139 | 1408 |  | 1 | 11 | 142 |
| 83 |  | malignant hematopoietic neoplasm |  | 0.7544 |  | 3 | 127 | 1338 |  | 0 | 23 | 214 |
| 84 |  | gastric ulcer |  | 0.7937 |  | 3 | 124 | 1279 |  | 0 | 26 | 271 |
| 85 |  | myelogenous leukemia |  | 0.8694 |  | 3 | 200 | 1969 |  | 0 | 10 | 101 |
| 86 |  | squamous-cell carcinoma of the lung | | 0.8699 |  | 3 | 202 | 1997 |  | 0 | 8 | 72 |
| 87 |  | diabetic nephropathy |  | 0.8711 |  | 3 | 143 | 1476 |  | 0 | 7 | 74 |
| 88 |  | pancreatic cancer |  | 0.8875 |  | 3 | 204 | 2009 |  | 0 | 6 | 61 |
| 89 |  | brain hemorrhage |  | 0.8910 |  | 2 | 108 | 1114 |  | 1 | 13 | 144 |
| 90 |  | osteoporosis |  | 0.8921 |  | 2 | 133 | 1352 |  | 1 | 17 | 195 |
| 91 |  | type 2 diabetes |  | 0.8943 |  | 2 | 128 | 1319 |  | 1 | 22 | 233 |
| 92 |  | duodenal ulcer |  | 0.8988 |  | 3 | 143 | 1484 |  | 0 | 7 | 66 |
| 93 |  | sepsis |  | 0.8991 |  | 3 | 104 | 1105 |  | 0 | 19 | 184 |
| 94 |  | aspiration |  | 0.8995 |  | 2 | 141 | 1446 |  | 1 | 9 | 106 |
| 95 |  | cholelithiasis |  | 0.9005 |  | 3 | 128 | 1326 |  | 0 | 22 | 224 |
| 96 |  | head and neck cancer |  | 0.9006 |  | 3 | 208 | 2048 |  | 0 | 2 | 21 |
| 97 |  | uterine cancer |  | 0.9122 |  | 2 | 84 | 912 |  | 0 | 2 | 19 |
| 98 |  | urinary tract infection |  | 0.9181 |  | 3 | 144 | 1495 |  | 0 | 6 | 57 |
| 99 |  | colon cancer |  | 0.9334 |  | 3 | 194 | 1922 |  | 0 | 16 | 150 |
| 100 |  | left ventricular hypertrophy |  | 0.9445 |  | 3 | 107 | 1118 |  | 0 | 43 | 430 |
| 101 |  | other tumors |  | 0.9545 |  | 3 | 209 | 2060 |  | 0 | 1 | 9 |
| 102 |  | presence of latent cancer |  | 0.9627 |  | 1 | 113 | 1101 |  | 1 | 17 | 185 |
| 103 |  | biliary tract cancer |  | 0.9830 |  | 3 | 204 | 2014 |  | 0 | 6 | 58 |
| 104 |  | aortic dissection |  | 0.9866 |  | 3 | 147 | 1517 |  | 0 | 3 | 30 |
|  |  |  |  |  |  |  |  |  |  |  |  |  |
|  |  |  |  |  |  |  |  |  |  |  |  |  |
| **^†^, comparison between heavy-smokers and other subjects; ^§^, comparison between heavy-smokers and light smokers; *, *p* < 0.05** | | | | | | |  |  |  |  |  |  |
