# Supplementary material for: Associations between the orexin (hypocretin) receptor 2 gene polymorphism Val308Ile and nicotine dependence in genome-wide and subsequent association studies
Source: Mol Brain. 2015 Aug 20;8:50. doi: 10.1186/s13041-015-0142-x (PMC4546081; doi:10.1186/s13041-015-0142-x)
Supplement: Additional file 12: Table S11. — Demographic data of patient subjects with clinical data related to smoking behavior. (DOC 77 kb) [file 13041_2015_142_MOESM12_ESM.doc]

| **Table S11. Demographic data of patient subjects with clinical data related to smoking behavior.** | | | | | | | |
| --- | --- | --- | --- | --- | --- | --- | --- |
|  |  |  |  |  |  |  |  |
|  |  | ***n*** | **Minimum** | **Maximum** | **Mean** | **SD** | **Median** |
|  |  |  |  |  |  |  |  |
| **All subjects** |  | 999 |  |  |  |  |  |
| male |  | 606 |  |  |  |  |  |
| female |  | 383 |  |  |  |  |  |
| unknown |  | 10 |  |  |  |  |  |
|  |  |  |  |  |  |  |  |
| **Age (years)** |  | 999 | 60 | 94 | 73.58 | 5.86 | 73.00 |
|  |  |  |  |  |  |  |  |
| **Height (cm)** |  | 996 | 130 | 180 | 156.86 | 8.69 | 158.00 |
|  |  |  |  |  |  |  |  |
| **Weight (kg)** |  | 995 | 30 | 101 | 54.75 | 9.97 | 54.00 |
|  |  |  |  |  |  |  |  |
| **Smoking status** |  | 999 |  |  |  |  |  |
| current smokers |  | 130 |  |  |  |  |  |
| ex-smokers |  | 392 |  |  |  |  |  |
| never-smokers |  | 477 |  |  |  |  |  |
|  |  |  |  |  |  |  |  |
| **Smoking behavior** |  | 522 |  |  |  |  |  |
| Smoking period (years) |  | 520 | 1.00 | 68.00 | 40.50 | 14.40 | 44.00 |
| FTND |  | 513 | 0.00 | 10.00 | 3.52 | 2.21 | 3.00 |
| TDS |  | 512 | 0.00 | 9.00 | 2.93 | 2.18 | 3.00 |
| CPD |  | 520 | 1.00 | 100.00 | 21.44 | 13.42 | 20.00 |
| NTC |  | 124 | 0.00 | 6.00 | 1.40 | 1.57 | 1.00 |
| NTE |  | 384 | 1.00 | 6.00 | 2.11 | 1.54 | 1.00 |
|  |  |  |  |  |  |  |  |
|  |  |  |  |  |  |  |  |
| FTND, Fagerstrӧm Test for Nicotine Dependence; TDS, Tobacco Dependence Screener; CPD, cigarettes smoked per day; | | | | | | |  |
| NTC, number of trials for smoking cessation in current-smokers; NTE, number of trials for smoking cessation in ex-smokers | | | | | | |  |
