# Supplementary figures and images for: Associations between the orexin (hypocretin) receptor 2 gene polymorphism Val308Ile and nicotine dependence in genome-wide and subsequent association studies
Source: Mol Brain. 2015 Aug 20;8:50. doi: 10.1186/s13041-015-0142-x (PMC4546081; doi:10.1186/s13041-015-0142-x)

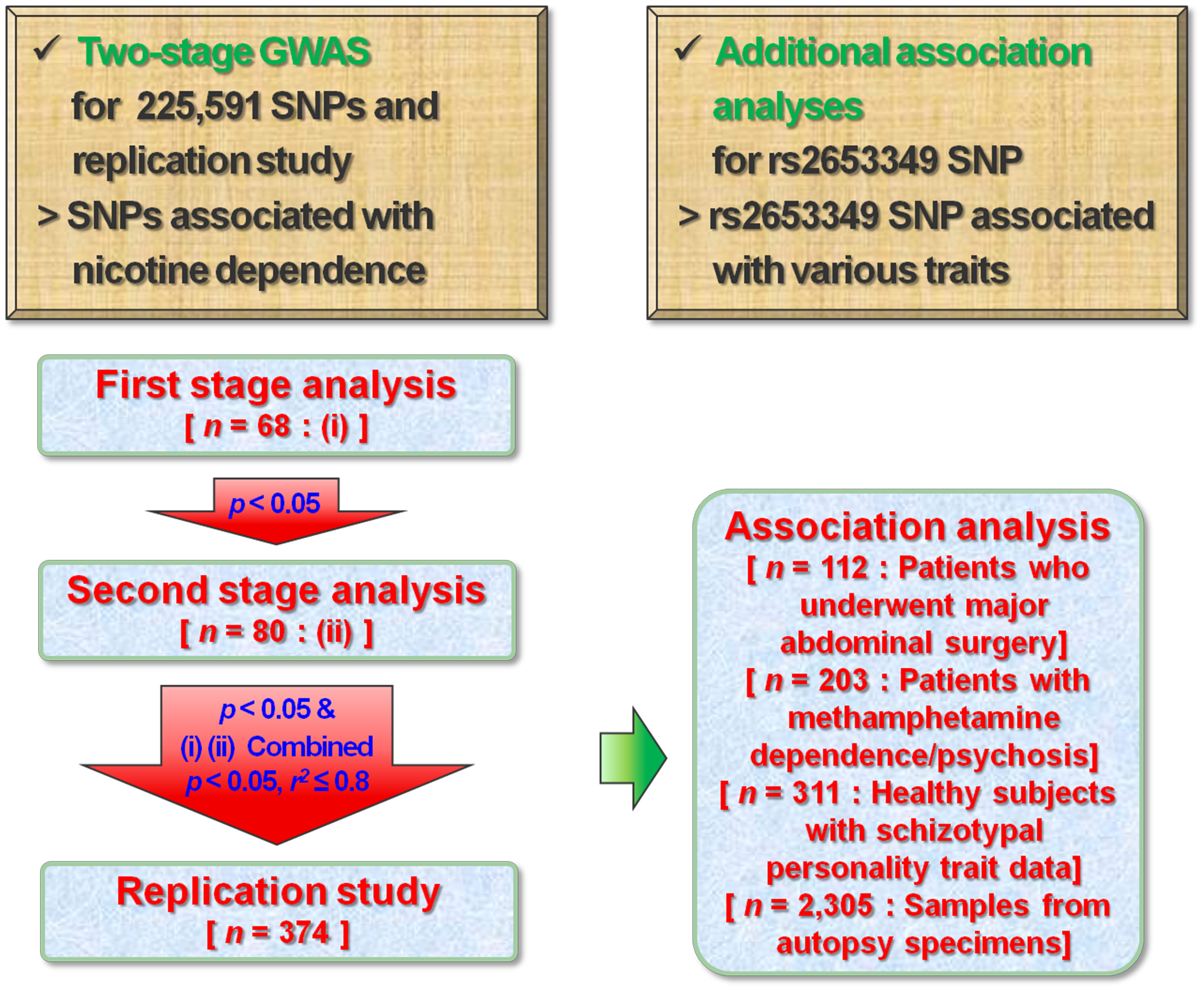

Supplement: Additional file 13: Figure S1. — Schematic illustration of the two-stage GWAS. Candidate SNPs associated with nicotine dependence in Japanese were selected. (TIFF 4809 kb) [file 13041_2015_142_MOESM13_ESM.tif]

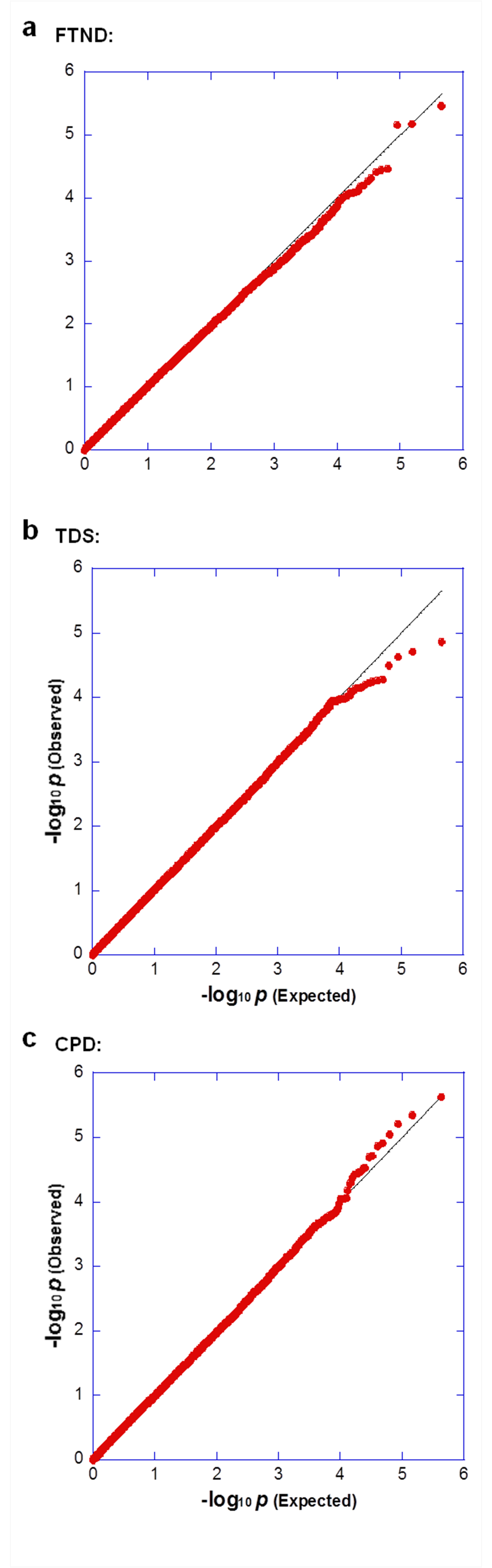

Supplement: Additional file 15: Figure S2. — Log quantile-quantile (QQ) p-value plot as a result of the genome-wide association study for the combined samples. (a) Plot of the results from the FTND. (b) Plot of the results from the TDS. (c) Plot of the results from the CPD. (TIFF 411 kb) [file 13041_2015_142_MOESM15_ESM.tif]
