# Supplementary material for: Associations between the orexin (hypocretin) receptor 2 gene polymorphism Val308Ile and nicotine dependence in genome-wide and subsequent association studies
Source: Mol Brain. 2015 Aug 20;8:50. doi: 10.1186/s13041-015-0142-x (PMC4546081; doi:10.1186/s13041-015-0142-x)
Supplement: Additional file 14: Table S12. — Distribution of genotypes of the rs2653349 and rs726016 SNPs and results of tests for Hardy-Weinberg equilibrium. (DOC 69 kb) [file 13041_2015_142_MOESM14_ESM.doc]

| **Table S12. Distribution of genotypes of the rs2653349 and rs726016 SNPs and results of tests for Hardy-Weinberg equilibrium.** | | | | | | | | | | | |
| --- | --- | --- | --- | --- | --- | --- | --- | --- | --- | --- | --- |
|  |  |  |  |  |  |  |  |  |  |  |  |
| **Sample cohort** | **SNP** | ***n*** | **Genotype** | | |  | **Allele frequency** | |  | ***χ^2^*** | ***p*** |
|  |  |  | A/A | A/B | B/B |  | A | B |  |  |  |
|  |  |  |  |  |  |  |  |  |  |  |  |
| **Smoking behavior** | rs2653349 | 513 | 0 | 58 | 455 |  | 0.057 | 0.943 |  | 1.842 | 0.175 |
|  | rs726016 | 521 | 6 | 128 | 387 |  | 0.134 | 0.866 |  | 1.645 | 0.200 |
|  |  |  |  |  |  |  |  |  |  |  |  |
| **Abdominal** | rs2653349 | 112 | 0 | 13 | 99 |  | 0.058 | 0.942 |  | 0.425 | 0.514 |
|  |  |  |  |  |  |  |  |  |  |  |  |
| **METH** | rs2653349 | 202 | 0 | 20 | 182 |  | 0.050 | 0.950 |  | 0.548 | 0.459 |
|  |  |  |  |  |  |  |  |  |  |  |  |
| **Schizotypal** | rs2653349 | 311 | 0 | 24 | 287 |  | 0.039 | 0.961 |  | 0.501 | 0.479 |
|  |  |  |  |  |  |  |  |  |  |  |  |
| **Autopsy specimen** | rs2653349 | 2287 | 3 | 210 | 2074 |  | 0.047 | 0.953 |  | 0.953 | 0.329 |
|  |  |  |  |  |  |  |  |  |  |  |  |
|  |  |  |  |  |  |  |  |  |  |  |  |
| *n*, the number of samples; A/A, homozygote for the major allele in each SNP; A/B, heterozygote for the major allele in each SNP; B/B, homozygote for the minor allele in each SNP; Smoking behavior, patients with smoking behavior data; Abdominal, patients who underwent major abdominal surgery; METH, patients with methamphetamine dependence/psychosis; Schizotypal, healthy subjects with schizotypal personality trait data; Autopsy specimen, samples from postmortem autopsy specimens. | | | | | | | | | | | |
